# Quantifying Dense Multicomponent Slurries with In-Line ATR-FTIR and Raman Spectroscopies: A Hanford Case Study

**DOI:** 10.1021/acs.iecr.3c01249

**Published:** 2023-09-21

**Authors:** Rupanjali Prasad, Steven H. Crouse, Ronald W. Rousseau, Martha A. Grover

**Affiliations:** School of Chemical and Biomolecular Engineering, Georgia Institute of Technology, Atlanta, Georgia 30332, United States

## Abstract

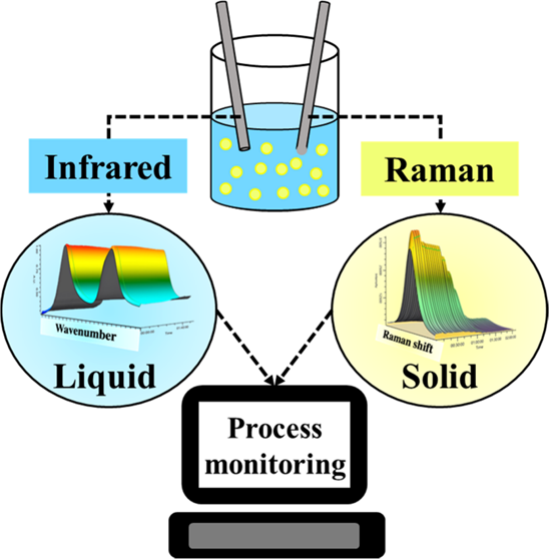

The multiphase nature of slurries can make them difficult to process and monitor in real time. For example, the nuclear waste slurries present at the Hanford site in Washington State are multicomponent, multiphase, and inhomogeneous. Current analytical techniques for analyzing radioactive waste at Hanford rely on laboratory results from an on-site analytical laboratory, which can delay processing speed and create exposure risks for workers. However, in-line probes can provide an alternative route to collect the necessary composition information. In the present work, Raman spectroscopy and attenuated total reflectance–Fourier transform infrared (ATR-FTIR) spectroscopy are tested on simulants of nuclear waste slurries containing up to 23.2 wt % solids. We observe ATR-FTIR spectroscopy to be effective in measuring the solution phase of the studied slurry systems (3.52% mean percent error), while Raman spectroscopy provides information about the suspended solids in the slurry system (18.21% mean percent error). In-line measurement of multicomponent solids typical of nuclear waste processing has been previously unreported. The composition of both the solution and solid phases is vital in ensuring stable glass formulation and effective disposal of nuclear waste at Hanford. Raman and ATR-FTIR spectroscopies can provide a safer and faster alternative for acquiring compositional information on nuclear waste slurries.

## 1 Introduction

The Hanford site in Washington State contains multiphase and multicomponent radioactive waste that poses a significant environmental and anthropological hazard for both current and future generations.^1,2^ Of the 56 million gallons of legacy nuclear waste at Hanford, approximately 1 million gallons have leaked from underground tanks. To address environmental issues, the United States Department of Energy is acting both to clean up the released waste and to immobilize waste that is still in containment. The waste still in the underground tanks will be separated into low-activity waste (LAW) and high-level waste (HLW) before being vitrified into borosilicate glass via the addition of glass-forming chemicals (GFCs).^3^ However, the addition of GFCs depends on the chemical composition of the incoming nuclear waste. “Grab-sampling” (analytical measurements using ion chromatography and inductively coupled plasma mass spectrometry) can provide the required measurements of stream composition.^4^ However, sampling in this manner provides a radioactive exposure risk for workers and can delay downstream decision making until analytical laboratory results are provided. Process analytical technologies (PAT), specifically Raman spectroscopy and attenuated total reflectance–Fourier transform infrared (ATR-FTIR) spectroscopy, provide alternatives to the currently planned “grab-sampling” technique. However, the use of ATR-FTIR and Raman spectroscopies has been poorly studied in multicomponent systems with concentrated solids. Because of the variety of real systems that contain solid particulate matter, the results presented here are important for operations at Hanford and have a bearing on real-time monitoring in many fields.

Optical spectroscopy has been applied as a PAT tool in the pharmaceutical, food, and mining industries. ATR-FTIR spectroscopy primarily measures the solution phase due to a short irradiation depth of infrared light, while Raman spectroscopy (not in an ATR configuration) interrogates both the solution phase and suspended solid particles. However, as seen in Table 1, most applications of these spectroscopies have been limited to systems with concentrations of suspended solids up to 5 wt % or to binary components at higher solid loadings.^5^ Although other spectroscopies such as X-ray fluorescence (XRF) and near-infrared (NIR) spectroscopy have been used in the mining industry to investigate systems containing 21 wt % solids (Table 1), the application of vibrational spectroscopy for monitoring multicomponent slurries with high solids content is scarce. Specifically in the nuclear field, Raman spectroscopy and ATR-FTIR spectroscopy have been shown effective in identifying and quantifying molecular species in solution. Despite complex solution behavior and overlapping spectral bands, progress has been made in the analysis of streams of nuclear waste through preprocessing techniques,^6^ physical modeling,^7^ and use of multiple excitation wavelengths.^8^ However, much of this work has been done by analyzing optically transparent solutions without high concentrations of suspended solids.

**Table 1:** Application of Spectroscopic Techniques for Quantification of Slurries in Various Industries

compound	application	suspended solids (wt %)	probes	description	ref
l-glutamic acid	pharmaceutical	∼4.5 wt %	ATR-FTIR	• real-time concentration data required during cooling crystallization for maintaining control over supersaturation.	(15)
				• partial least squares (PLS) regression method used for chemometric modeling.	
paracetamol, l-glutamic acid	pharmaceutical	3.4 wt %	Raman	• the application of linear and nonlinear models for estimating solution concentration and slurry density was discussed.	(16)
				• different polymorphs of the compounds were also considered while estimating solution concentration and slurry density.	
				• nonlinear models showed better prediction ability.	
salicylic acid	pharmaceutical	12 wt %	ATR UV–vis and NIR	• kinetic modeling of dissolution and crystallization of salicylic acid from ethanol–water solution has been studied.	(17)
				• ATR UV–vis is used for estimating the concentration of solute in solution while NIR is used to obtain information about the solid phase.	
L-menthol	pharmaceutical	0.4–2.9 wt %	Raman	• solid densities along with liquid–liquid suspension densities were monitored using Raman spectroscopy.	(18)
				• Raman spectroscopy was applied to detect liquid–liquid phase separation (LLPS) during oiling out crystallization.	
compound A and D (names not mentioned)	pharmaceutical	2.4 wt %	ATR-FTIR, FBRM, and Raman spectroscopy	• four partial least squares (PLS) regression models were developed to convert the spectroscopic data to slurry density, solute concentration in solution, diastereomeric composition of the crystals, and percent composition of diastereomer in solution.	(19)
				• model in which slurry density was calculated using IR spectral data showed slightly better prediction accuracy.	
ortho-aminobenzoic acid	pigments, dyes, perfumes and pharmaceutical	29 wt %	ATR UV–vis, FBRM, PVM, and Raman spectroscopy	• the effect of temperature, crystal size, and solute and solid concentration on the Raman spectra was studied.	(5)
				• a multivariate approach using PLS regression could predict the solid density with reasonable accuracy.	
sodium carbonate and sodium bicarbonate	industrial crystallization	5 wt %	Raman spectroscopy	• used support vector regression for separating information on solution and suspended solids.	(20)
mineral flotation slurry	mining industry	2.5–6.3 wt %	visible and near-infrared reflectance (VNIR) spectroscopy	• a change in the composition of the mineral slurry was found to have a greater impact on the spectra than the amount of solid content in the slurry.	(21)
				• a combination of VNIR spectroscopy and X-ray fluorescence (XRF) gave a continuous online estimation of the slurry contents.	
pig slurry	anaerobic digestion	∼2.3 wt %	offline near-infrared spectroscopy (NIRS)	• moving window principal component analysis (MWPCA) was used to monitor the lipid, organic, and protein concentrations.	(22)
				• the MWPCA model developed with offline NIRS spectral data was applied for the detection of mechanical disturbances in the anaerobic digestor.	
mineral flotation slurry	mining industry	21 wt %	X-ray fluorescence (XRF) and near-infrared (NIR) spectroscopy	• minerals such as scheelite, wolframite, calcite, and fluorite in a mineral flotation slurry were monitored via XRF, NIR spectroscopy, and fusion of XRF and NIR spectroscopy.	(23)
				• data fusion improved the quantitative prediction of the minerals when compared to quantification based on only NIR or only XRF.	

For HLW processing at Hanford, the currently planned process is designed for 20 wt % insoluble solids.^9^ For the currently planned LAW process, the addition of glass-forming chemicals will create a slurry with roughly 22–33 wt % insoluble solids.^10^ Since the application of PAT to monitor nuclear waste slurries has received scant attention, this work investigates the applicability of in-line probes to analyze slurries representative of nuclear waste containing up to 400 g of insoluble solids/kg solvent (23.2 wt %). Measuring the solids concentration of slurries with Raman spectroscopy presents challenges because of the strongly absorbing and scattering properties of most slurries, in addition to competing fluorescence effects.^11,12^

The present work focuses on a specific scenario in waste processing at Hanford. Specifically, monitoring the composition of nuclear waste feeds within the melter feed preparation vessel (MFPV) was used as a case study of nuclear waste containing suspended solids. The MFPV will have a similar purpose in both LAW and HLW processing: that is, glass-forming chemicals are added to the nuclear waste in the MFPV tank before eventually being transported to the melter. Compositional measurements of the MFPV are planned to verify proper compositions for a durable glass form before being melted.^13^ The analytical measurements of the slurry in the MFPV vessel represent a hold point during HLW vitrification, indicating that the waste will not be further processed until concentration measurements are obtained. Therefore, the implementation of in-line probes may offer advantages by facilitating faster downstream decision making and mitigating the risk associated with grab samples. In addition to the specific study of nuclear waste entering the MFPV, much of the work presented here is also applicable to other processing instances that have multicomponent suspended solids.^14^

## 2 Methods

### 2.1 Instrumentation

Measurements were collected at a 100 mL scale in a Mettler Toledo (MT) OptiMax reactor (250 mL) installed with a pitched-blade agitator (Alloy C-22, downward, ø 45 mm) fitted with the following in situ devices: a Raman probe, a pH probe, a temperature probe, and an attenuated total reflectance–Fourier transform infrared (ATR-FTIR) probe (Figure S1a). A Teflon vessel was used due to the high basicity of the solution. Raman spectra were recorded with a Mettler Toledo ReactRaman 785 instrument using a 785 nm laser at 300 mW power, 1 s exposure time, 10 averaged spectra, and a spectral resolution of 6 cm^–1^. The Raman probe tip is a ball-probe configuration leading to a focal point of about 200 μm from the probe surface.^24,25^ Infrared spectra were recorded with a Mettler Toledo ReactIR 10 instrument with a diamond probe tip and a spectral resolution of 8 cm^–1^. The ATR design limits the penetration depth of the infrared radiation to ∼2 μm.^26^ Data acquisition was performed by using iC Raman and iC IR software from Mettler Toledo. Fouling of the ATR-FTIR probe was observed in the presence of silicates (Figure S2). To minimize the effect of fouling, the ATR-FTIR probe was cleaned before each measurement. No fouling was observed on the Raman probe; the difference in the fouling behavior between Raman and ATR-FTIR probe tips may be caused by a difference in material (sapphire for Raman and diamond for ATR-FTIR) or geometry (convex for Raman and concave for ATR-FTIR).

### 2.2 Composition

The system is based on the 5.6 M Na^+^ low-activity waste (LAW) pretreatment system simulant (sodium salts)^27^ combined with simulated glass-forming chemical (GFC) recipes composed predominantly of metal oxides and silicates.^10^Figure S1b shows a slurry obtained after the addition of GFCs with the simulants (900 g GFCs/kg water). The LAW simulants comprise water, sodium hydroxide, and seven sodium salts: nitrate, nitrite, carbonate, sulfate, phosphate, oxalate, and acetate. The glass-forming chemical compositions were formulated and provided by the Savannah River National Laboratory.^10^ The GFC simulants comprise five insoluble silicates: silica, kyanite, wollastonite, olivine, and zircon; four metal oxides: hematite, rutile, tin oxide, and zinc oxide; and four additional soluble species: vanadium pentoxide, boric acid, sucrose, and lithium carbonate. The concentration ranges for all species are listed in Tables S1–S3. All experiments were conducted at 3 M NaOH to simulate the basic conditions expected at the Hanford Waste Treatment Plant. At this molality of sodium hydroxide, the pH remained above 13 for all experiments, which was verified with an *in-situ* pH probe. The experiments were temperature-controlled to 25 °C and stirred at 400 rpm to maintain a suspension of solids inside the apparatus. The experiments were designed using the MATLAB (2022b) random number generator to randomly design compositions within the bounds of each species. Additional details about the validity of the experimental design are discussed in Section S1 in the Supporting Information.

### 2.3 Data Preprocessing

All preprocessing steps were performed with Python 3.9.12. The python code and the data set can be found on Github: https://github.com/magrover/multicomponent-slurry-quantification. ATR-FTIR data were narrowed to a range of 900–1800 cm^–1^ for quantification. This range contains all solution peaks provided by the instrument. A Savitzky–Golay filter^28,29^ was used to differentiate the ATR-FTIR spectra with respect to wavenumber with five filter points, a second-order polynomial, and first derivative. The publicly available package, SciPy, was used to perform Savitzky–Golay filtering. The Raman data were narrowed to a range of 100–1700 cm^–1^ for quantification. The range contains all observable peaks for the insoluble species. No derivative was taken for the Raman spectra because of the correlation between baseline and some solid species, although the effect of first-derivative Savitzky–Golay filtering may improve model performance and reproducibility and is shown in Figure S3 and Table S4 in the Supporting Information.

### 2.4 Regression Model

A linear spectra-to-concentration relationship is expected with a Raman probe in an ideal system.^26,30^ The quantitative basis for Raman spectroscopy is given as
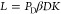
1where *L* is a measure of intensity [photons/(sr·c·s)], *P*_D_ is the power of the laser [photons/(cm^2^·s)], β is the differential Raman cross section [cm^2^/(mol·sr)], *D* is the density of Raman scattering molecules [molecules/cm^3^], and *K* is a dimensionless geometric factor accounting for detection angle.^12,30^Equation 1 shows that when *P*_D_β*K* is constant, the detected Raman scattering depends linearly on the density of scattering molecules. Attenuation or absorption can affect the Raman bands of optically dense samples, which might be seen in a slurry with suspended solids.^12,30,31^ A total of 34 experimental replicates were performed to demonstrate the experimental reproducibility of ball-probe Raman spectroscopy measurements in the presence of solids (Figures S4 and S5). There was a 1.24% mean difference between the original and replicate spectra (Section S4).

For the ATR-FTIR probe, the Beer–Lambert law is expected to apply to solution phase measurements, with little interference from suspended solids.^26^ The Beer–Lambert law is given as
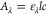
2where *A*_λ_ is absorbance at a particular wavelength, ε_λ_ is the molar absorptivity at a particular wavelength [L/(mol·cm)], *l* is the effective path length [cm], and *c* is the species′ concentrations [mol/L].

Partial least squares regression (PLSR)^32,33^ was chosen as the spectra-to-composition model in this work. PLSR has been used for the analysis of both nuclear waste solutions^27,34−36^ and pharmaceutical slurries.^16,19,30^ The scikit-learn package (version 1.0.2) implementation of PLSR was used for all quantifications in this work. An additional description of PLSR utilized in this work is in Section S5.

As part of the analysis in Sections 3.4 and 3.5, mixture spectra are predicted from the gravimetrically measured concentrations of both dissolved salts and suspended solids. An indirect classical least squares (ICLS) method is used for visualizing what spectra “should” look like for Raman or ATR-FTIR-based eqs 1 and 2.^37^ For ICLS predictions, all of the spectra are mean-centered. Linear references are determined from the mixture data by fitting the experimental spectra of mixtures with known concentrations. The calculated references are used in conjunction with eqs 1 and 2 to predict spectra with gravimetrically measured concentrations. In Sections 3.4 and 3.5, predicted spectra are used to show deviations from the assumed linear models.

## 3 Results and Discussion

### 3.1 Solution Measurements with Probes

**Figure 1:**
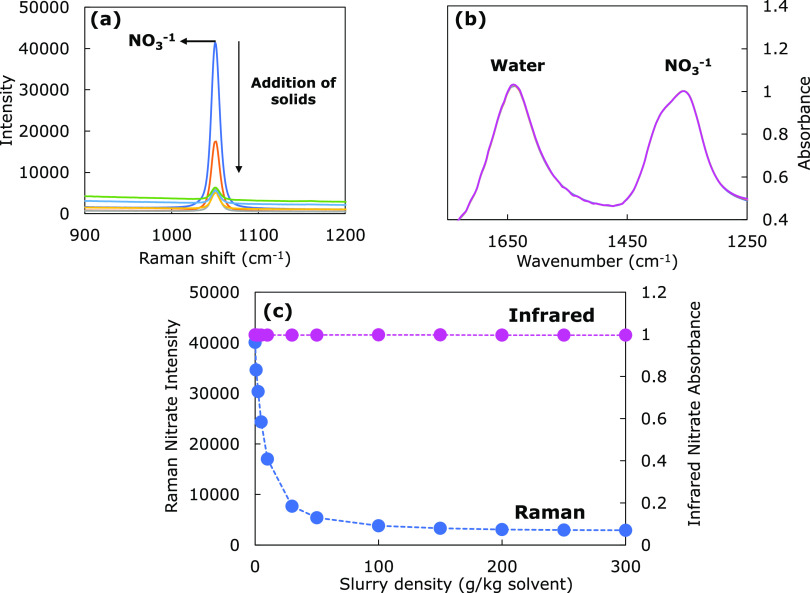
Spectra for (a) Raman and (b) ATR-FTIR spectroscopy at different concentrations of suspended solid particles and (c) attenuation of the nitrate peak with increasing solids concentration.

The analysis of the studied slurry system is enabled by the complementary capabilities of the Raman probe and the ATR-FTIR probe. Figure 1 demonstrates a ternary system consisting of soluble sodium nitrate, insoluble silica, and a 3 M NaOH solution. Response profiles can be seen for Raman (Figure 1a) and ATR-FTIR (Figure 1b) probes when the concentration of suspended solids is increased. Notably, the ATR-FTIR instrument can detect soluble NO_3_^–^ anions with no apparent dependence on solids concentration in the solution (Figure 1b). This result matches other published research indicating that the infrared radiation does not appreciably contact suspended solids given the shallow penetration depth of the ATR mode of operation.^26,38^ The Raman probe, however, is in a ball-probe configuration with a sapphire lens and has a path length approximately 2 orders of magnitude greater than a probe in ATR configuration.^24^ Because of the increased path length, the Raman probe may be affected by the optical density of the slurry. In Figure 1a and Figure 1c, the Raman probe shows a reduced NO_3_^–^ signal intensity with an increase in solid concentration, suggesting that Raman spectroscopy may not be effective at measuring the solution phase at high solid concentrations. However, this does not preclude Raman spectroscopy from providing information about the solid phase of the slurry, which is not provided by probes in the ATR configuration.

### 3.2 ATR-FTIR and Raman Spectroscopy Analysis of Individual GFC Components Dispersed in Alkaline Media

The components of glass-forming chemicals were grouped into three categories based on their solubility in basic solution (solubility results shown in Table S5 and Figure S6): insoluble species (silica and other silicates such as kyanite, wollastonite, olivine, and zircon), metal oxides (hematite, rutile, tin oxide, and zinc oxide), and soluble species (vanadium pentoxide, boric acid, and lithium carbonate). Solubilities were estimated using inductively coupled plasma (ICP) over 10 days (Section S6).

#### 3.2.1 Soluble Species

**Figure 2:**
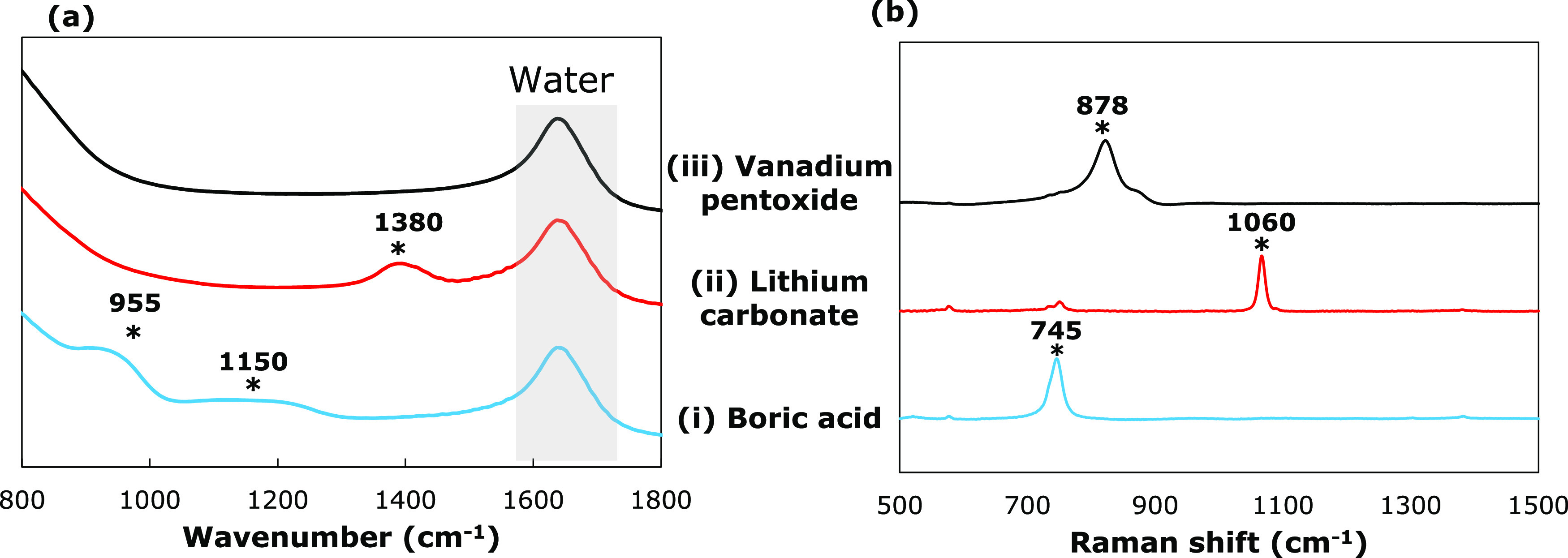
(a) FTIR and baseline-corrected (b) Raman spectra of soluble GFC components in a basic 3 M NaOH solution: (i) Boric acid (B(OH)_3_), (ii) lithium carbonate (Li_2_(CO)_3_), and (iii) vanadium pentoxide (V_2_O_5_). The characteristic peaks have been marked with an ‘*’, and their corresponding wavenumbers have been listed.

The FTIR and Raman reference spectra of soluble GFC components (vanadium pentoxide, boric acid, and lithium carbonate) in a 3 M NaOH solution (pH ≥ 13) are shown in Figure 2. All FTIR spectra in Figure 2a have a water band at approximately 1640 cm^–1^ corresponding to the O–H bending band of water (ν_2_ mode).^39^ Each of the spectra in Figure 2 corresponds to a concentration of 1 m, except for lithium carbonate (Li_2_CO_3_). The solubility of lithium carbonate is less than 1 m; hence, the Raman and FTIR spectra correspond to a saturated solution of the salt (0.304 m, determined in this work) at 25 °C. The dissolution of boric acid (B(OH)_3_) at a high pH (greater than 13) is given by the following reaction
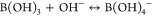
3

At a high pH, boric acid mainly dissociates into the borate ion,^40,41^ B(OH)_4_^–^, whose presence can be further corroborated by examining the spectrum of boric acid solution in Figure 2a(i). The FTIR spectrum exhibits spectral features at 1150 and 950 cm^–1^. The broad peak at 1150 cm^–1^ corresponds to the B–O–H in-plane bending, while the peak at 950 cm^–1^ is caused by the B–O asymmetric stretching vibrations in the borate ion.^40,41^ The corresponding Raman peak for the borate ion in Figure 2b(i) is observed at 745 cm^–1^, representing the total symmetrical vibrations.^42^

Lithium carbonate shows significant dissolution and dissociation (eq 4) under alkaline conditions into carbonate (CO_3_^2–^) ions.
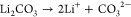
4

The FTIR and Raman spectra of lithium carbonate in 3 M NaOH solution are shown in Figure 2a(ii) and 2b(ii), respectively. The FTIR spectrum exhibits a strong peak at 1380 cm^–1^, which originates due to the C–O asymmetrical in-plane stretch of the carbonate ion (CO_3_^2–^).^39^ The corresponding Raman spectrum also has a sharp peak around 1060 cm^–1^ arising due to the C–O symmetric stretching vibrations.^43^

Vanadium pentoxide (V_2_O_5_) is an amphoteric oxide and is soluble in strong alkaline solutions to form metavanadate (VO_3_^–^) and orthovanadate (VO_4_^3–^) ions
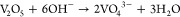
5
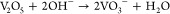
6

At pH ≥ 13, the main ion present in the solution is the orthovanadate ion^44^ (eq 5), which does not appear on the FTIR spectrum (Figure 2a(iii)) but is Raman-active. The presence of the orthovanadate ion can be deduced by the spectral bands in the Raman spectrum (Figure 2b(iii)). The Raman spectrum shows two bands at 878 and 825 cm^–1^. The strongest band at 878 cm^–1^ is due to the symmetric stretching of the VO_3_ units, while the 825 cm^–1^ is associated with the symmetric stretching vibrations of the VO_2_ units.^45^

#### 3.2.2 Insoluble Species

**Figure 3:**
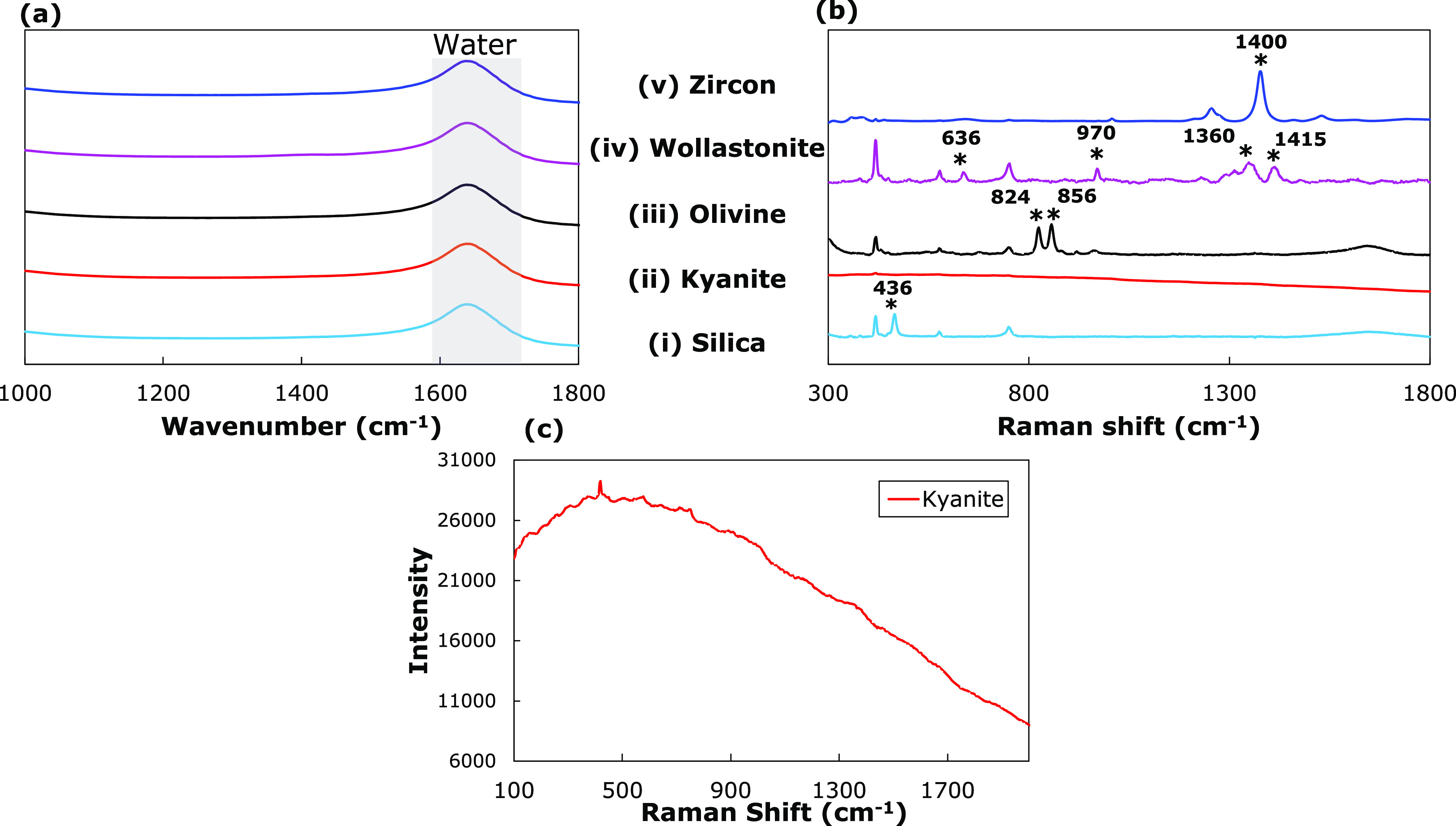
Highest-peak normalized (a) ATR-FTIR spectra of filtered silicate solution and baseline-corrected (b) Raman spectra of suspended silicates (50 g/kg water) in a basic 3 M NaOH solution: Silica (SiO_2_), Kyanite (Al_2_SiO_5_), Olivine (Mg_2_SiO_4_), Wollastonite (CaSiO_3_), and Zircon (ZrSiO_4_). (c) Unnormalized Kyanite spectrum showing broad fluorescence background. The Raman peaks corresponding to the solids have been marked with an ‘*’, and their corresponding wavenumbers have been listed.

The FTIR and Raman references for the insoluble species were obtained by adding 5 g (50 g/kg water) of a single type of solid (silica, kyanite, olivine, wollastonite, or zircon) to a 3 M NaOH solution and stirring overnight. The silicates were observed to deposit on the ATR-FTIR probe window (Figure S2b). Therefore, FTIR reference spectra for silicates were obtained by pipetting around 5 mL of the solution containing solid particles, followed by centrifugation and filtration by passing the supernatant through a syringe filter (0.22 μm pore size) to ensure that all solid particles were removed from the solution before measuring their FTIR spectra (Figure 3a). The reference Raman spectra, on the other hand, were obtained by analyzing the slurry (containing solids suspended via mixing) with a Raman probe (Figure 3b,c).

The FTIR spectra exhibit a single peak at 1640 cm^–1^ (Figure 3a), which is attributed to the O–H bending vibrations of the water molecule.^39^ Apart from the bending vibrations of the O–H from water at 1640 cm^–1^, no other spectral features were observed in the FTIR spectra (Figure 3a) for the five studied silicates. Similarly, no peaks were observed in the Raman spectra of the filtered solutions (data not shown). This again demonstrates negligible solubility, as shown by ICP results in Section 3.2.

The reference Raman spectra of solids (silica and silicates) in a 3 M NaOH solution are shown in Figure 3b. Three peaks are present in every measurement located at 419, 577, and 751 cm^–1^, due to the sapphire tip of the probe. The Raman spectrum of silica (SiO_2_) in Figure 3b(i) has a sharp peak at 436 cm^–1^, which arises due to the symmetric stretching vibrations of the Si–O–Si units.^46,47^ Similar Si–O–Si stretching interactions in olivine occur as a doublet at 824 and 856 cm^–1^ (Figure 3b(iii)). Both features arise from coupled symmetric and asymmetric stretching vibrational modes of the SiO_4_ tetrahedral units.^48^ Raman spectra of wollastonite show multiple features in the spectral region of 600–1500 cm^–1^ (Figure 3b(iv)). The peak at 636 cm^–1^ is attributed to the Si–O–Si bending vibrations, whereas the bands at 970, 1360, and around 1415 cm^–1^ are due to Si–O stretching vibrations.^49^ Zircon also exhibits a sharp peak at 1400 cm^–1^ arising from the Si–O stretching vibrations. The Raman spectrum of kyanite (Al_2_SiO_5_) does not show strongly resolved peaks. However, the kyanite spectrum has a signature broad shape centered around 500 cm^–1^ (Figure 3c) that is also observed in the spectra of GFC mixtures.

#### 3.2.3 Metal Oxides

The FTIR and Raman spectra of various oxides constituting the GFCs are shown in Figures S7a and S7b, respectively. The slurries probed using ATR-FTIR and Raman contain around 5 g of the solid compound dispersed in a 3 M NaOH solution (50 g/kg water). Except for the peak at 1640 cm^–1^, which is attributable to the O–H bending vibrations in the water molecule, no spectral features are observed in the FTIR spectra (Figure S7a). However, ICP data (Table S5) show that some oxides, particularly zinc oxide (ZnO), are significantly soluble at a high pH. Dissolution information combined with the observed spectra of Figure S7a indicates that these oxides are not IR-active in the wavenumber range studied or that the sensitivity of the probe is not high enough to detect dissolved concentrations. The corresponding Raman spectra of the solid compounds exhibit several peaks for suspended particles of rutile (TiO_2_, Figure S7b(i)) and hematite (Fe_2_O_3_, Figure S7b(iv)) that can be used for identification of the compounds in a mixture. Peaks at 143, 447, and 612 cm^–1^ are observed in the Raman spectra of rutile (Figure S7b(i)) corresponding to the B_1g_, E_g_, and A_1g_ vibrational modes, respectively. The Raman spectra of hematite have peaks at 227, 293, 418, and 610 cm^–1^ (Figure S7b(iv)). While the peak at 227 cm^–1^ represents the A_1g_ phonon band mode, the peaks at 293, 418, and 610 cm^–1^ are attributed to the *E*_g_ mode vibrations. These bands involve the displacement of both iron and oxygen within Fe(O)_6_ octahedral units.^50,51^ The other oxides, zinc oxide and tin oxide (SnO_2_), do not have any visible spectral features in the measured Raman spectra except for background fluorescence (Figure S7b(ii),(iii)).

### 3.3 Quantification of Slurries

**Figure 4:**
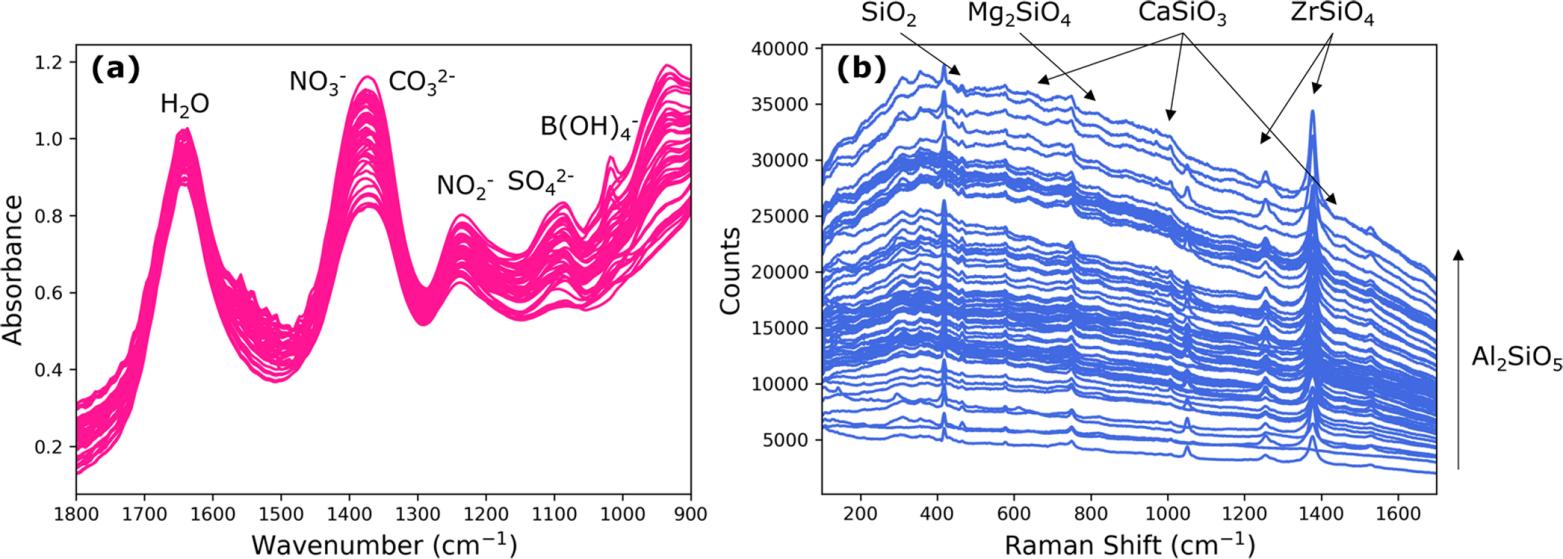
Overlapped (a) ATR-FTIR and (b) Raman spectra before separation into training and testing sets.

ATR-FTIR was used to quantify the concentration of dissolved molecular species in slurries comprising glass-forming chemicals in nuclear waste simulants. Based on the system studied, the most abundant (and therefore process-relevant) soluble species were quantified with ATR-FTIR:^27,52^ NO_3_^–^, NO_2_^–^, CO_3_^2–^, and SO_4_^3–^. In addition, borate (B(OH)_4_^–^) was chosen to quantify in the solution phase based on boric acid having high solubility and the distinguishable FTIR peak intensity shown in Section 3.2. Overlapping spectra of all 48 ATR-FTIR experiments are shown in Figure 4a, along with the peaks of the soluble species quantified. Two of the studied soluble species are contributed from components in the glass-forming chemicals: lithium carbonate (yielding a soluble carbonate anion) and boric acid (yielding a soluble borate anion). The dissolution and dissociation of lithium carbonate (Section S7) imply that the carbonate anion has two sources in the studied slurries: sodium carbonate (from waste simulants) and lithium carbonate (from solid GFCs). Lithium carbonate dissolution was approximated as 0.304 m (pure lithium carbonate solubility in a 3 M sodium hydroxide solution at 25 °C) to calibrate the PLSR model with gravimetric measurements of total carbonate concentration. The carbonate dissolution model resulted in improved carbonate quantification as demonstrated in Figure S8 and is used in carbonate quantification in this work.

Raman spectroscopy was used to quantify the concentration of suspended solids in slurries of glass-forming chemicals in a simulant solution. Solid silicate species: silica, kyanite, olivine, wollastonite, and zircon, were studied due to their abundance in the solid GFC mixtures compared to other solid compounds and their limited dissolution. Overlapping Raman spectra of all 66 unique experiments at different solids concentrations are shown in Figure 4b.

### 3.4 Solution Phase Quantification with ATR-FTIR Spectroscopy

**Figure 5:**
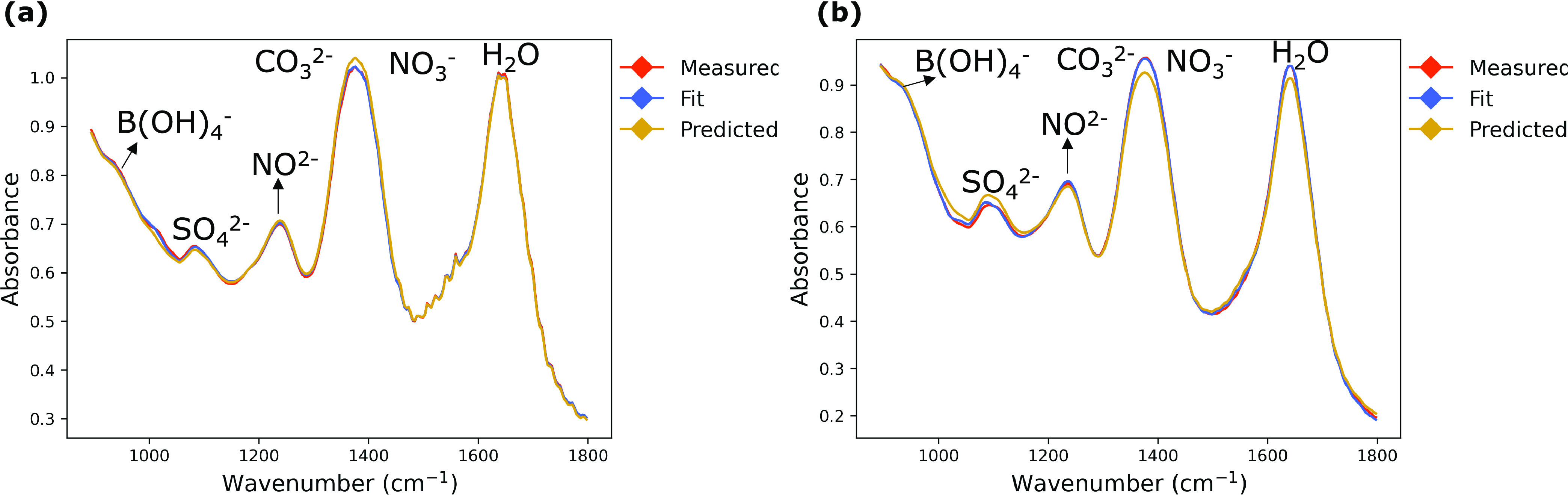
Two different ATR-FTIR experiments (a, b) fit using indirect classical least squares (ICLS) and predicted using gravimetrically measured masses.

Analysis of the FTIR spectra can be performed by comparing the measured spectra to predictions from the Beer–Lambert law (eq 2). In Figure 5a,b, two measured experimental spectra (shown in red) are fit with indirect classical least squares (least squares fit shown in blue). Based on the known solution concentrations and fit references, the application of the Beer–Lambert law predicts mixture spectra (shown in yellow). Table 2 quantifies the differences in the spectra that may be difficult to observe based on the spectra alone (Figure 5).

**Table 2:** Predicted Concentrations from ATR-FTIR Spectra from Figure 5 Using a PLSR Model

concentrations (mol/kg solvent)	nitrate	nitrite	carbonate	sulfate	borate
Figure 5a predicted	0.863	0.711	0.517	0.025	0.305
Figure 5a gravimetric	0.883	0.719	0.538	0.026	0.304
Figure 5b predicted	0.877	0.827	0.560	0.073	0.862
Figure 5b gravimetric	0.879	0.818	0.522	0.078	0.852

From Figure 5a, the predicted spectrum (based on known masses added) overpredicts the measured spectrum at the nitrate/carbonate peak (1400 cm^–1^), suggesting that the measured spectrum has a negative deviation from the Beer–Lambert law. A negative deviation is reflected in the quantification of the measured spectrum of Figure 5a in Table 2, as both nitrate and carbonate are underpredicted based on the measured spectra. Another deviation from the Beer–Lambert law can be observed in Figure 5b. The predicted spectrum overpredicts the measured spectrum at the sulfate peak (1100 cm^–1^), suggesting that the measured spectrum has a negative deviation from the Beer–Lambert law. Table 2, again, shows that a PLSR model underpredicts sulfate based on the experimental spectrum in Figure 5b. The size of the deviations shown in the two experiments in Table 2, with a mean deviation of ±0.114 mol/kg solvent, shows general agreement between the Beer–Lambert law and measured ATR-FTIR spectra in slurry conditions.

**Figure 6:**
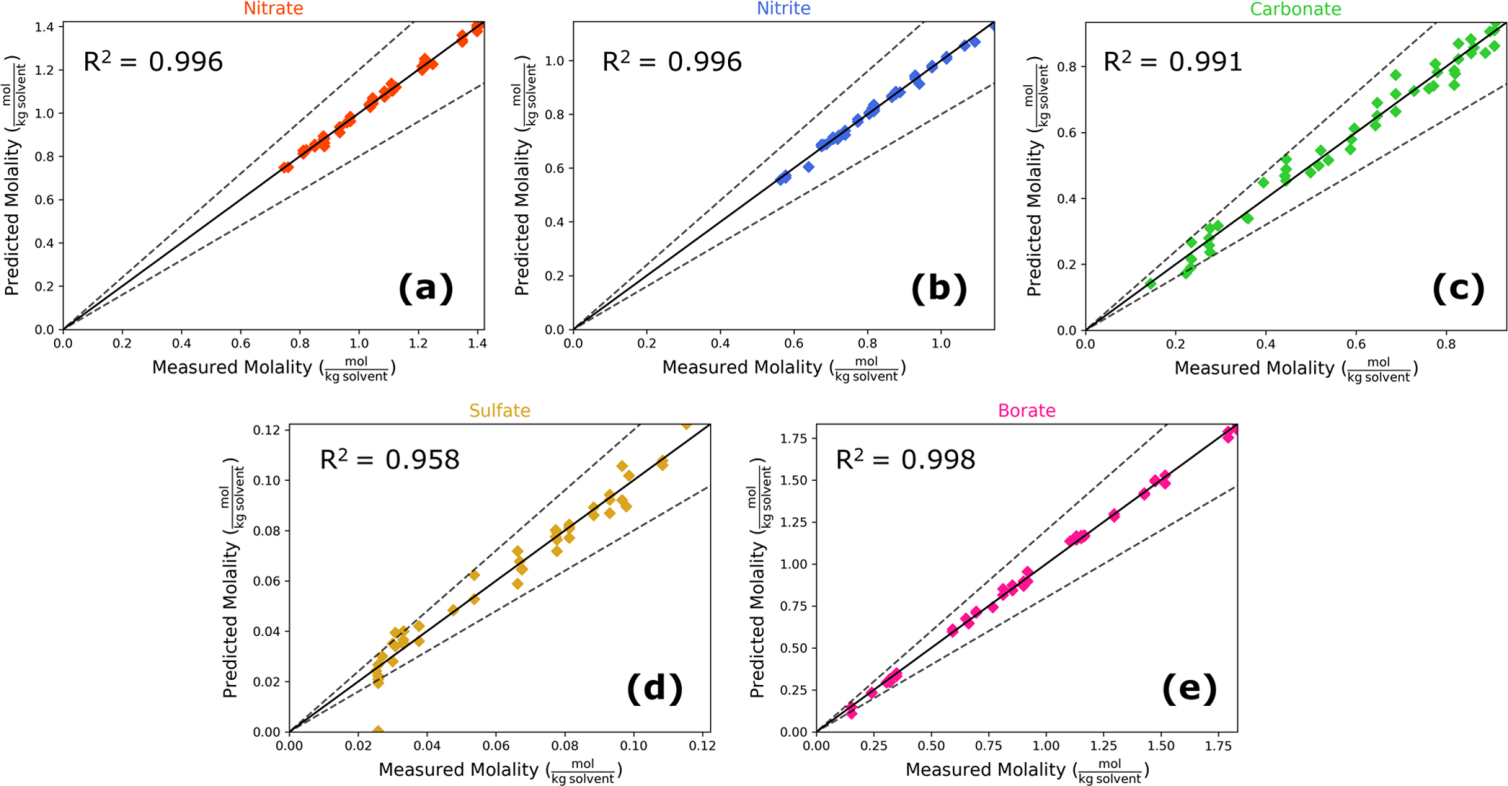
Parity plots (showing ±20% bounds) for soluble anion quantification in the simulant slurry using ATR-FTIR spectra input into a PLSR model with 15 latent variables for (a) nitrate, (b) nitrite, (c) carbonate, (d) sulfate, and (e) borate.

The quantification of soluble anions is shown in Figure 6, with each data point having a unique concentration of dissolved analytes. Bounds of ±20% are motivated by measurement accuracy specifications at the WTP Process Control Data Quality Objectives^53^ and are shown in Figure 6 by dashed lines for reference. Nitrate, nitrite, and borate are quantified most accurately with prediction *R*^2^ values over 0.995 over the tested concentration ranges. Notably, nitrate and nitrite do not exceed a ±20% limit over process-relevant concentrations in this study. Carbonate, sulfate, and borate exceed the ±20% bound, primarily at lower concentrations. One sulfate measurement produced a particularly poor prediction (shown by the sulfate prediction at 0 mol/kg solvent). This particular experiment had near the minimum amount of both boric acid and sulfate. Both boric acid and sulfate being at low concentrations may have resulted in the poor sulfate quantification at that point, particularly since sulfate and boric acid have overlapping peaks in that wavenumber range and sulfate has a relatively large limit of detection compared to its tested concentrations. Residual plots can be found in Figure S9. Four accuracy metrics—mean prediction accuracies, RMSE, 95% confidence bounds, and mean percent error, are listed in Table 3.

**Table 3:** Anion Quantification Accuracy with ATR-FTIR

metric	nitrate	nitrite	carbonate	sulfate	borate
mean absolute error (mol/kg solvent)	0.0101	0.0074	0.0148	0.0040	0.0150
root mean squared error (mol/kg solvent)	0.0127	0.0091	0.0201	0.0058	0.0196
one-sided 95% confidence interval (mol/kg solvent)	0.0277	0.0183	0.0362	0.0087	0.0422
mean percent error (%)	0.96	0.94	3.78	9.37	2.54

### 3.5 Solid Phase Quantification with Raman Spectroscopy

**Figure 7:**
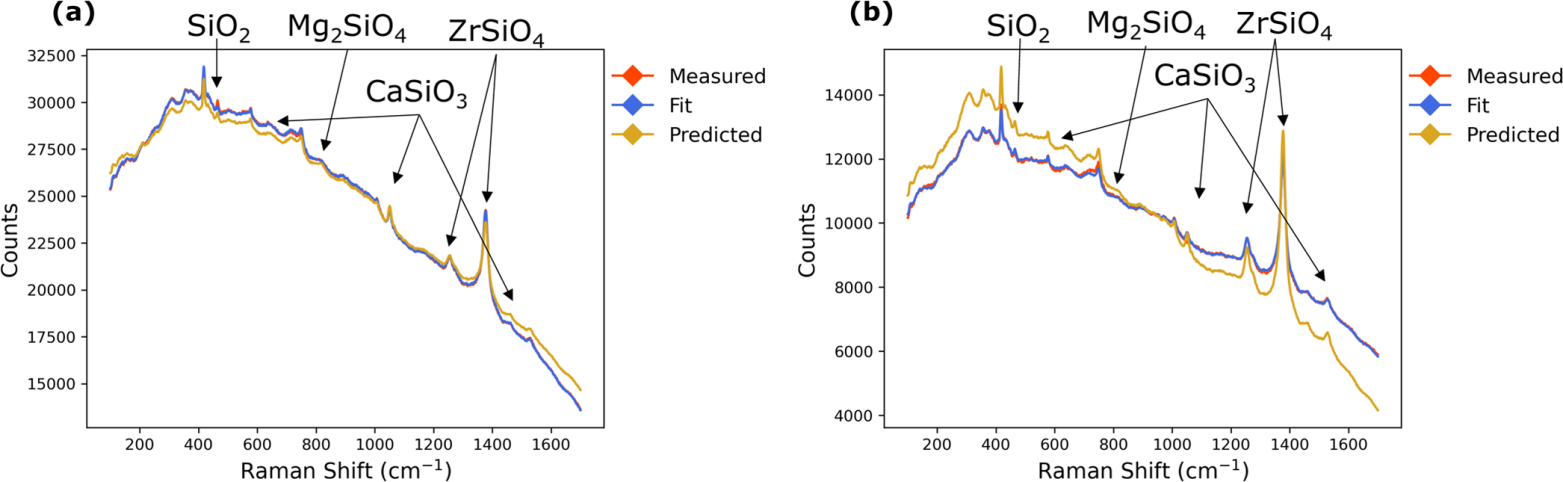
Two different Raman experiments (a, b) fit using indirect classical least squares (ICLS) and predicted using gravimetrically measured masses.

Analysis of the Raman spectra can be performed by comparing measured spectra to spectra that a linearity assumption predicts in eq 1. In Figure 7a,b, two measured experimental spectra (shown in red) are fit with indirect classical least squares (least squares fit shown in blue) to compute linear references for kyanite, wollastonite, silica, olivine, and zircon. Predicted spectra (shown in yellow) are calculated from known, gravimetrically measured slurry concentrations and fit references. Table 4 quantifies the differences in spectra that are evident in Figure 7.

**Table 4:** Prediction Accuracy of Raman Spectra from Figure 7 Using a PLSR Model

concentrations (g/kg solvent)	kyanite	wollastonite	olivine	silica	zircon
Figure 7a predicted	81.58	25.57	33.91	146.61	19.71
Figure 7a gravimetric	81.41	16.97	41.67	142.03	18.67
Figure 7b predicted	57.96	39.33	18.31	120.80	25.50
Figure 7b gravimetric	64.69	41.96	11.09	135.68	32.10

Figure 7 shows that the experimental (measured) and fitted spectra match each other closely. In Figure 7b, the predicted spectrum does not match the measured spectrum as closely as in Figure 7a. The predicted spectrum overpredicts the slope of the background in Figure 7b, which correlates closely with kyanite concentrations. This would indicate a negative deviation from the Beer–Lambert law for Kyanite in this experiment. The deviation results in underprediction of kyanite in this sample, as shown in Table 4. Another example can be seen with the sharp peak at 1400 cm^–1^ corresponding to zircon, which is much more prominent in the linearly predicted (yellow curve) spectra than in the experimental spectra (red curve) in Figure 7b. This would similarly suggest a negative deviation from linearity for zircon and likewise appears as an underprediction in Table 4.

**Figure 8:**
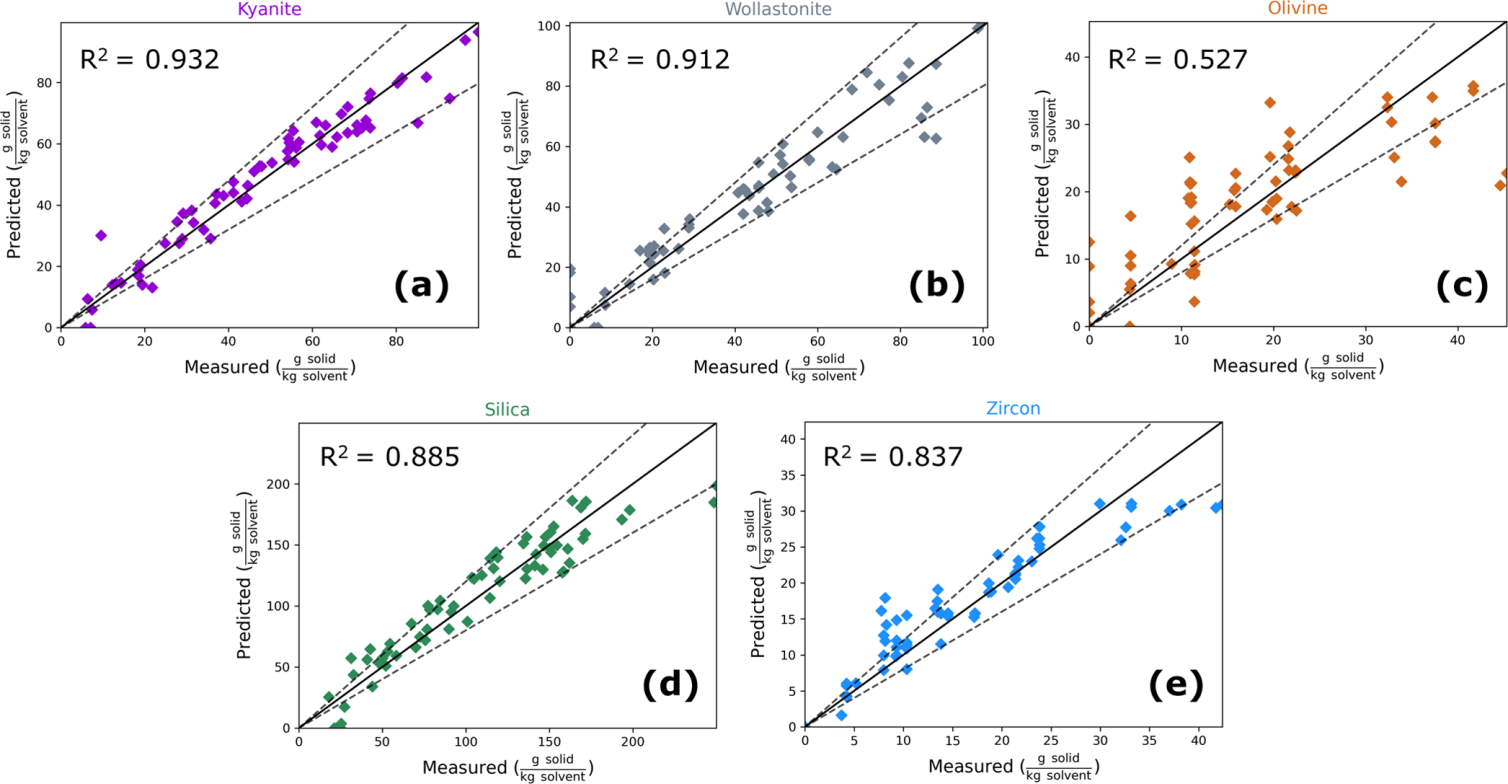
Parity plots (showing ±20% bounds) of major insoluble silicate quantification using Raman spectra input into a PLSR model with 10 latent variables for (a) kyanite, (b) wollastonite, (c) olivine, (d) silica, and (e) zircon.

Parity plots showing the quantification of silicate solids concentration with a ±20% bound in dashed lines are shown in Figure 8. Notably, kyanite and wollastonite show the best prediction performance with *R*^2^ values of 0.932 and 0.912 respectively. Silica and zircon have slightly less accurate quantification, with *R*^2^ values of 0.885 and 0.837, respectively. Olivine is predicted with the least accuracy, showing an *R*^2^ value of 0.527. Residual plots can be found in Figure S10. A pattern can be seen in the parity plots of Figure 8, where the PLSR model appears to underpredict true solid content at high solids content. This effect is most prominent in zircon quantification, although it may be present in the predictions of other species as well. One possible explanation for this apparent patterning may be the loss of quantification linearity at high solids content.

The poor quantification of olivine may be attributed to its low abundance in the slurry and its obscured spectral features. Zircon, despite its similarly low slurry concentration, is highly Raman-active in the region studied with a prominent peak at 1378 cm^–1^. Due to the low signal-to-noise ratio of Raman in these slurries, olivine quantification may be improved by including measurements with higher proportions of olivine. Four accuracy metrics—mean prediction accuracies, RMSE, 95% confidence bounds, and mean percent error—are listed in Table 5. The independence of the quantified chemical species is depicted in Figures S11 and S12.

**Table 5:** Solid Quantification Accuracy with Raman

metric	kyanite	wollastonite	olivine	silica	zircon
mean absolute error (g/kg solvent)	4.57	5.66	5.20	13.53	2.52
root mean squared error (g/kg solvent)	6.04	7.88	6.95	17.29	3.68
one-sided 95% confidence interval (g/kg solvent)	9.15	16.18	12.80	28.58	7.40
mean percent error (%)	16.5	16.7	39.4	18.2	21.4

## 4 Summary and Conclusions

Establishing accurate models and the conceptual feasibility of monitoring dense slurry solutions is important for nuclear waste management and many other systems. The present work shows that instrumentation employing ATR-FTIR technology can accurately estimate the concentrations of key solutes in the liquid (solution phase) portion of a slurry containing a concentration of suspended solids (up to 23.2 wt %) with a mean accuracy of 3.52%. Companion results using Raman spectroscopy facilitate the ability to distinguish and quantify different species suspended in the solid phase with a mean accuracy of 18.21% for the four most abundant and spectroscopically active silicates. While this work demonstrates the feasibility of Raman and ATR-FTIR spectroscopies for monitoring slurries typical of nuclear waste processing, the wide application breadth of these instruments does not limit the bearing of these results to the highlighted application.

## Supporting Information Available

This material is available free of charge via the Internet at http://pubs.acs.org/. The Supporting Information is available free of charge at https://pubs.acs.org/doi/10.1021/acs.iecr.3c01249.

Details on experimental methods; ATR-FTIR probe tip fouling; effect of preprocessing conditions such as first derivative and Savitzky–Golay filtering on the quantification of solids from Raman spectra; experimental analysis describing the reproducibility of the Raman spectra; partial least squares regression model; solubility studies of various components in alkaline media; IR and Raman reference spectra of oxides; carbonate solubility model to account for two sources of carbonate anion in solution; parity plots depicting carbonate quantification with and without the carbonate solubility model; predicted concentrations from ATR-FTIR and Raman spectra using PLSR model; and residual analysis (PDF)
