# Supplementary material for: Quantifying Dense Multicomponent Slurries with In-Line ATR-FTIR and Raman Spectroscopies: A Hanford Case Study
Source: Ind Eng Chem Res. 2023 Sep 21;62(39):15962–73. doi: 10.1021/acs.iecr.3c01249 (PMC10557100; doi:10.1021/acs.iecr.3c01249)
Supplement: Supplementary file 1 — ie3c01249_si_001.pdf [file ie3c01249_si_001.pdf]

Supplementary Information

Quantifying Dense Multicomponent Slurries with In-line ATR-FTIR and Raman Spectroscopy: A Hanford case study

Rupanjali Prasad[ǂ], Steven H. Crouse[ǂ], Ronald W. Rousseau, Martha A. Grover[*]

School of Chemical and Biomolecular Engineering, Georgia institute of Technology, Atlanta, Georgia 30332, United States

*Email: martha.grover@chbe.gatech.edu

[ǂ]R.P. and S.C. contributed equally to this paper

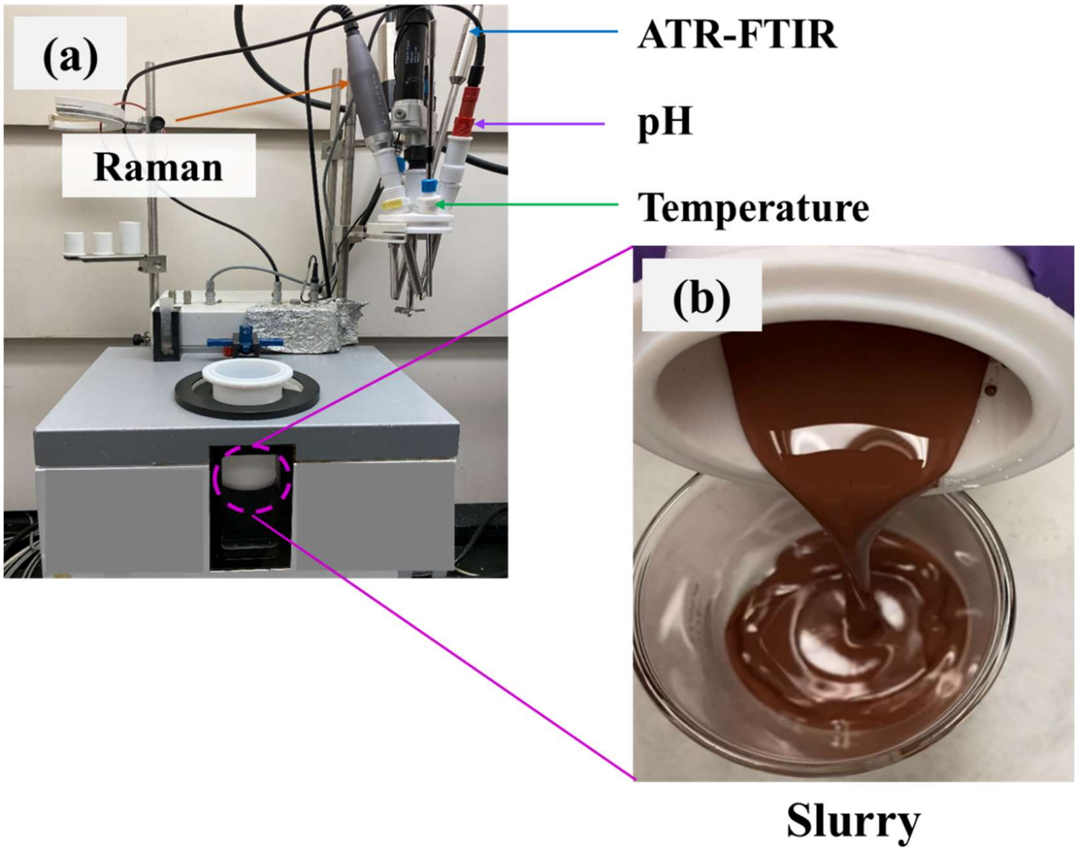

Figure S1. (a) Photograph of the OptiMax reactor setup with *in-situ* probes: Raman, ATR-FTIR, temperature, and pH, (b) a close-up view of the dense slurry studied in this work.

Section S1. Design of Experiments

The experimental space was designed by creating component-wise lower and upper bounds based on reported nuclear waste simulants.[1,2] Species are bounded by concentrations expected in nuclear waste streams at the Hanford Waste Treatment Plant. Individual experiments were conducted by randomly sampling from this experimental space using a pseudo-random uniform distribution in MATLAB 2021a. Experiments were performed in batches ranging from 6 to 12 step-wise samples. 48 unique samples (at different concentrations) were collected for ATR-FTIR, and 66 unique samples (at different concentrations) were collected for Raman. Verification of the independence of the species are shown in Section S9. When silicates were present, solid particles deposited on the ATR-FTIR probe during data collection. To eliminate the spectral bands caused by depositing solids, the ATR-FTIR probe was cleaned before every measurement containing silicates to ensure reliable spectra. Every sample was allowed to equilibrate before the ATR-FTIR

probe was cleaned and a measurement was taken. Data showing this deposition is provided in Section S2. All solid species are nominally 325-mesh or less (corresponding to a diameter of 45 µm or less). The ranges of added concentrations are listed below for insoluble and partially soluble species in Tables S1 and S2, and soluble species in Table S3. The solubility for these species in 3 m NaOH is reported in Section S6.

Table S1 - Range of solid concentrations tested in experiments by addition of glass forming chemicals (insoluble components in 3 m NaOH).

Species	*Formula*	*Maximum Solid Concentration (g / kg water)*	*Minimum Solid Concentration (g / kg water)*
Kyanite	Al_2SiO_5	99.6	5.9
Wollastonite	$CaSiO_3$	99.8	0
Olivine	Mg_2SiO_4	45.3	0
Silica	SiO_2	249.9	18
Zircon	$ZrSiO_4$	42.4	0
Hematite	Fe_2O_3	9.4	0
Rutile	TiO_2	1.2	0
Tin Oxide	SnO_2	12.7	0
Total (Single Experiment)	-	401.8	53.1

Table S2 - Range of solids composing glass forming chemicals with intermediate solubility measured in 3 m NaOH.

Species	*Formula*	*Maximum Addition (g / kg water)*	*Minimum Addition (g / kg water)*
Vanadium Pentoxide	V_2O_5	31.9	0
Zinc Oxide	ZnO	22.5	0
Sucrose	$C_{12}H_{22}O_{11}$	53.7	4.7

Table S3 - Range of dissolved anions present in the nuclear waste simulants studied (soluble components in 3 m NaOH).

Species	*Formula*	*Maximum Molality (mol / kg water)*	*Minimum Molality (mol / kg water)*
Hydroxide	OH^-	2.98	2.94
Nitrate	NO_3^-	1.44	0.75
Nitrite	NO_2^-	1.16	0.56
Soluble Carbonate	CO^{2-}	0.96	0.14
Sulfate	SO_4^{2-}	0.13	0.03
Borate	$B(OH)_4^-$	2.84	0.15
Phosphate	PO_4^{3-}	0.07	0.02
Oxalate	$C_2O_4^{2-}$	0.01	0.00
Acetate	$C_2H_3O_2^-$	0.19	0.02
Sucrose	$C1_2H_{22}O_{11}$	0.24	0.01

Section S2. ATR-FTIR Probe Tip Cleaning

The silicates (silica, kyanite, wollastonite, olivine, and zircon) were observed to deposit on the ATR-FTIR probe at the basic conditions studied. This deposition appeared as a broad combination of peaks centered around 1100 cm^{-1} and spreading from 1000 cm^{-1} to 1200 cm^{-1}. In Fig. S3a, this peak can be seen as it appears in the IR spectrum and in Fig. S3b, images of the solid substance on part of the probe tip. Notably, the solids provide a peak but do not interfere with quantification of the solution phase (as shown by the unaltered water peak centered at 1640 cm^{-1}).

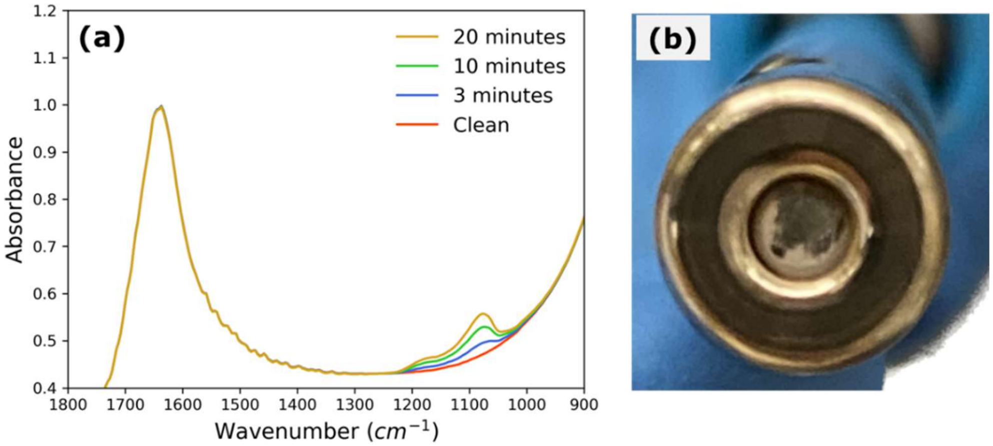

Figure S2. a) Deposition of solids on ATR-FTIR probe tip when placed in a 3 m NaOH solution with 10 g silica/kg solvent suspended silica, and b) image of the probe tip showing gradual buildup on the probe tip.

While the solid deposition was not observed to interfere with the solution phase measurements and could potentially be subtracted as a baseline in practice, the experimental procedure included cleaning the probe tip before every measurement to minimize unanticipated sources of variation and to ensure experimental consistency. Because of the slow buildup of solids on the probe tip, measurements were taken with 15 s scan time and immediately after reinserting probe into the solution. Detection and removal of solid contributions may be achieved through computational approaches such as blind source separation and Indirect Hard Modeling.[7,8] In addition, different materials or probe geometries may result in a less favorable surface for deposition. Solids were not found to attach to the Raman probe tip during experiments.

Section S3. First Derivative with Savitzky-Golay Filtering

Optionally, the first derivative could be taken of Raman spectra to minimize baseline shifts caused by fluorescence. This was done as a proof-of-concept with a Savitzky-Golay filter with a second order polynomial, 19 filter points, and a first derivative. For the noisy spectra utilized in this work, 11 filter points or fewer resulted in noisy derivative spectra. The spectra after filtering are shown below in Fig. S3 and quantification results are shown in Table S4. As can be seen in Table S4, quantification is improved after Savtizky-Golay filtering for wollastonite, olivine, silica, and zircon. Kyanite quantification is worse after filtering. These results show promise for the

Savitzky-Golay filter to preprocess noisy and varied Raman spectra for more accurate quantification.

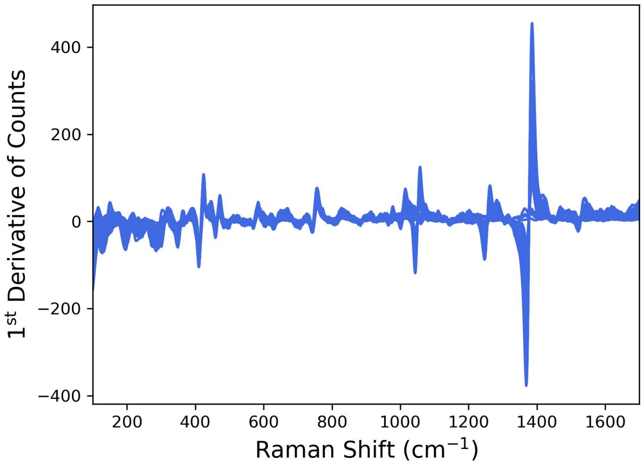

Figure S3. All Raman spectra of training and testing data when Savitzky-Golay filter has been applied.

Table S4. R^2 value of predictions for quantified insoluble species with and without Savitzky-Golay filtering

**Concentrations (g/kg solvent)**	***Kyanite***	***Wollastonite***	***Olivine***	***Silica***	***Zircon***
R^2 (No Filtering)	0.932	0.912	0.527	0.885	0.837
R^2 (Filtering, 1st Derivative)	0.912	0.916	0.770	0.905	0.863

Section S4. Experimental Replicates

A total of 34 replicate measurements were collected using slurries that were mixed and measured at least 11 (and no more than 15) days after the original data appearing in the main text (Fig. 4b). As can be seen in Fig. S4a, there is a baseline offset in some measurements, with the greatest difference in a measurement being 1495 counts (Fig. S4b). The average difference between the original measurement and its replicate across the 34 measurements is 239.6 counts (or 1.24% difference from original measurements). The difference between replicate measurements also shows high-frequency noise in addition to the baseline offset. The replicate data were also quantified via a PLSR model with and without Savitzky-Golay spectra preprocessing, shown in Figure S5. Original measurements only were used for model training. The Savitzky-Golay preprocessing appears to be more precise since a vertical offset appears in the predictions of the

raw spectra. This result agrees with the offset that can be seen in the replicate spectra. Based on these data, we conclude that both our experimental methodology and our Raman apparatus are robust to within a relatively small baseline offset for experimental replicates.

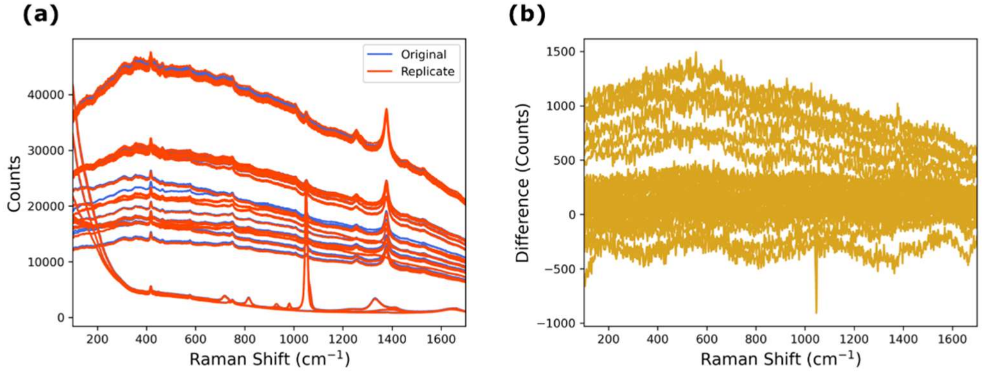

Figure S4. (a) Original spectra and replicate spectra collected for 34 replicate measurements ranging from 0 to 25 wt% solids, and (b) the difference between original and replicate spectra.

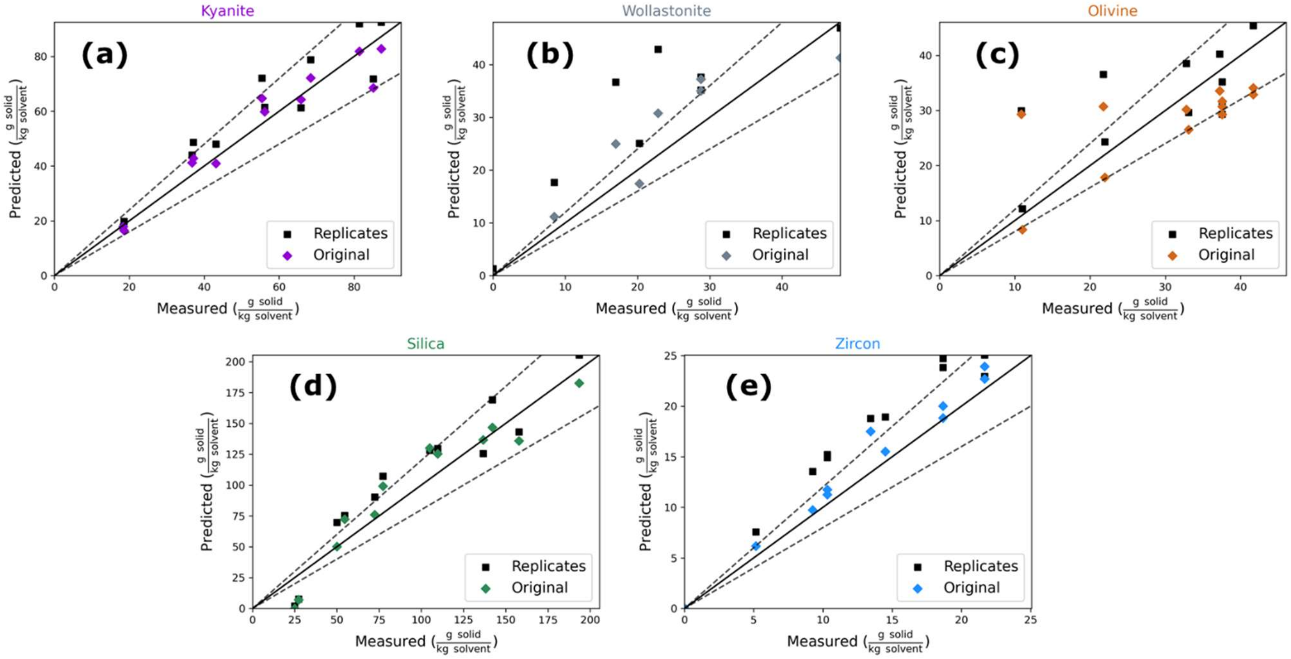

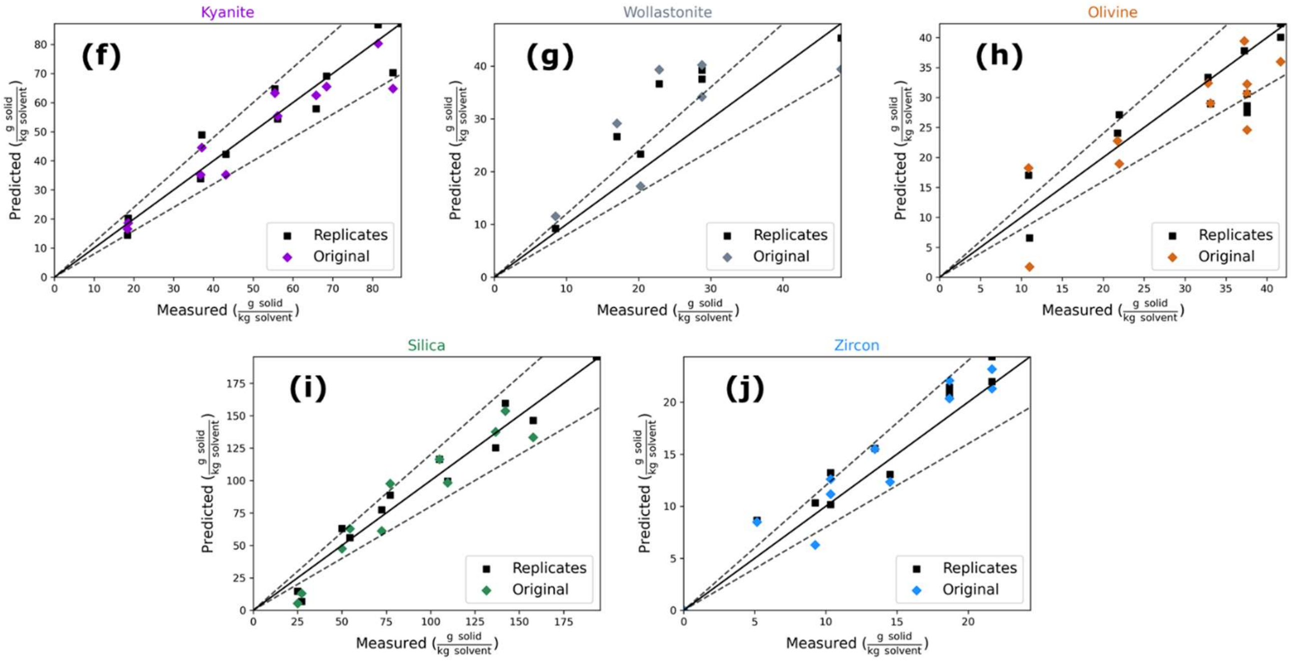

Figure S5. Parity plots showing PLSR predictions of original and replicate (a-e) raw spectra and (f-j) spectra passed through a 19-point Savitzky-Golay filter.

Section S5. Partial Least Squares Regression (PLSR)

Given a training data set, the relationship between inputs (X^{tr}) and outputs (Y^{tr}) can be used to develop a predictive model for new data. In this case, $X^{tr} \in \mathbb{R}^{N \times q}$ are spectral data and $Y^{tr} \in \mathbb{R}^{N \times p}$ are concentration data with N representing the number of training samples, q representing the features (discrete wavenumbers) in a single spectrum, and p representing the number of components (independent species) included in the calibration model. Motivated by the linearity of the Beer-Lambert Law and the analogous linearity of Raman Spectroscopy[3], a linear calibration model between X^{tr} and Y^{tr} can be represented by $X^{tr}B = Y^{tr}$ where $B \in \mathbb{R}^{q \times p}$ is a matrix of parameters.

Partial Least Squares Regression[4,5] is a projection-based based model that reduces the dimension of the input space by identifying underlying latent variables, Λ:

$$X^{tr}P = \Lambda^{tr} \tag{S1}$$

where $P \in \mathbb{R}^{q \times d}$ is a projection matrix and d is the reduced dimension ($d < q$). $\Lambda^{tr} \in \mathbb{R}^{N \times d}$ represents the matrix of latent variables for the training set. Multiple least-squares regression is

performed on the reduced set of latent variables to identify the calibration model. The objective function for the calibration model can be described by:

$$\text{minimize} \quad \left\| \Lambda^{tr} \tilde{b}_j - y_j^{tr} \right\|_2^2 \quad \text{by varying } \tilde{b}_j \in \mathbb{R}^d \quad \text{for } j = 1, \ldots, p \tag{S2}$$

where $P\tilde{b}_j = b_j$ and the minimized objective function is the squared error of the reduced variable (latent) model ($\Lambda^{tr}\tilde{b}_j$) and training output data (y_j^{tr}). The specific PLSR algorithm used was a scikit-learn implementation of PLS-2.

Four error metrics are used to quantify the quantitative accuracy of the spectra-to-composition PLSR models. These are Root Mean Square Error (RMSE), Mean Absolute Error (MAE), the one-sided 95% Confidence Interval (CI (95%)), and Mean Percent Error (MPE). The metrics are defined below in Equations S3-S6 for single species:

$$\text{RMSE} = \sqrt{\sum_{i=1}^{N} \frac{(y_i - \hat{y}_i)^2}{N}} \tag{S3}$$

$$\text{MAE} = \sum_{i=1}^{N} \frac{|y_i - \hat{y}_i|}{N} \tag{S4}$$

$$\text{CI (95\%)} = z \frac{s}{\sqrt{N}} \tag{S5}$$

$$\text{MPE} = \sum_{i=1}^{N} \frac{|y_i - \hat{y}_i|}{y_i} \times 100\% \tag{S6}$$

where y_i is the true concentration in experiment i, $\hat{y}_i$ is the predicted concentration in experiment i, N is the total number of experiments, s is the standard deviation of measured concentrations, and z is the confidence level value (±1.96 for 95%).

Training and Testing datasets are determined using a leave-one-out cross validation scheme. A single sample (test spectra) is estimated using a PLSR model trained on all other samples (training spectra), and then the process is repeated. Minimum Akaike Information Criterion (AIC) was used for determining the number of latent variables in each PLSR model.[6]

The Raman PLSR quantification model was determined to use 10 latent variables, while the ATR-FTIR PLSR quantification model was determined to use 15 components.

Section S6. Solubility of Solid GFC's in Alkaline Media

The spectra-to-composition model for solids is calibrated using measured addition weights of GFC mixtures to 3 m NaOH. An assumption underlying gravimetric calibration of the solid phase is that insignificant amounts of solids dissolve. Therefore, insignificant solubility of the target solids must be established before performing Raman measurements. As solid GFCs are composed of various silicates and oxides, the solubilities of these GFC-constituting compounds were determined experimentally due to limited reported data at high pH for many of the studied components. Approximately 5 g of GFCs were added to a 3 m NaOH solution (pH > 13) and the resulting slurry was stirred using a magnetic stirrer for 10 days. The solution-phase concentration of elemental species was monitored using inductively coupled plasma (ICP) spectroscopy. Fig. S6 depicts the solution phase concentration profiles of various elemental species undergoing dissolution in alkaline medium for a particular GFC mixture (GFC-1). The composition of GFC-1 along with the amount of each dissolved elemental species, determined using ICP, along with their source compound, is shown in Table S5. While the data obtained from ICP is elemental (such as vanadium, boron, zinc etc.), Table S5 shows the concentration of the respective molecular compounds (such as vanadium pentoxide, boric acid, zinc oxide etc.) inferred from the ICP analysis. Comparison between "dissolved" and "total" amounts provide a measure of the solubility of each component.

Table S5. Dissolved solids estimated from ICP measurements (± 5%) after ten days of dissolution, and total solids estimated from gravimetric measurements (± 0.2%); both given in (µg / g sample).

**Elemental species**	**Al**	**Ca**	**Mg**	**Si**	**Zr**	**Fe**	**Ti**	**Sn**	**Zn**	**B**	**Li**	**V**
**Source**	**Kyanite**	**Wollastonite**	**Olivine**	**Silica**	**Zircon**	**Hematite**	**Rutile**	**Tin Oxide**	**Zinc Oxide**	**Boric Acid**	**Lithium Carbonate**	**Vanadium Pentoxide**
Chemical formula	Al_2SiO_5	$CaSiO_3$	Mg_2SiO_4	SiO_2	$ZrSiO_4$	Fe_2O_3	TiO_2	SnO_2	ZnO	H_3BO_3	Li_2CO_3	V_2O_5
GFC-1 **Dissolved** (ICP)	335	398	-	518	-	-	-	3	828	18273	-	13080
GFC-1 **Total** (Mass)	33024	34885	-	34652	-	-	-	7907	930	18745	-	12791
GFC-2 **Dissolved** (ICP)	118	88		610				2	5219	14790	16309	11559
GFC-2 **Total** (Mass)	8874	8968	-	78731	-	-	-	-	6986	13594	15388	10384

The concentrations of all species were monitored for 10 days to determine which species constituting GFCs were highly soluble. According to the ICP results, a steady state was achieved within a day for all species except for silicon, whose solution concentration after 10 days was found to be 150 μg/g solution (Fig. S2). The dissolution of silicon does not significantly affect the amount of suspended undissolved silicate species; less than 0.15% of all molecules containing silicon dissolve in GFC-1 over 10 days, which is less than the precision of the gravimetric measurement. Vanadium (vanadium pentoxide) displayed the highest concentration (Table S5), followed by zinc (zinc oxide), lithium (lithium carbonate), and boron (boric acid). The results in Table S5 indicate that the solution was undersaturated for vanadium and lithium, as complete dissolution of their corresponding source compounds was observed. For compounds such as zinc oxide, the solution appeared to have reached saturation, as the dissolved amount was less than the total amount added in solution. Furthermore, the solubilities at basic conditions are considered negligible for aluminum (kyanite), calcium (wollastonite), and tin (tin oxide). Notably, the poor solubility of elemental silicon bounds the dissolution of the silicates in GFC-1 (kyanite, wollastonite, and silica) to a combined 150 (μg dissolved silicon /g sample) over 10 days for the solutions tested.

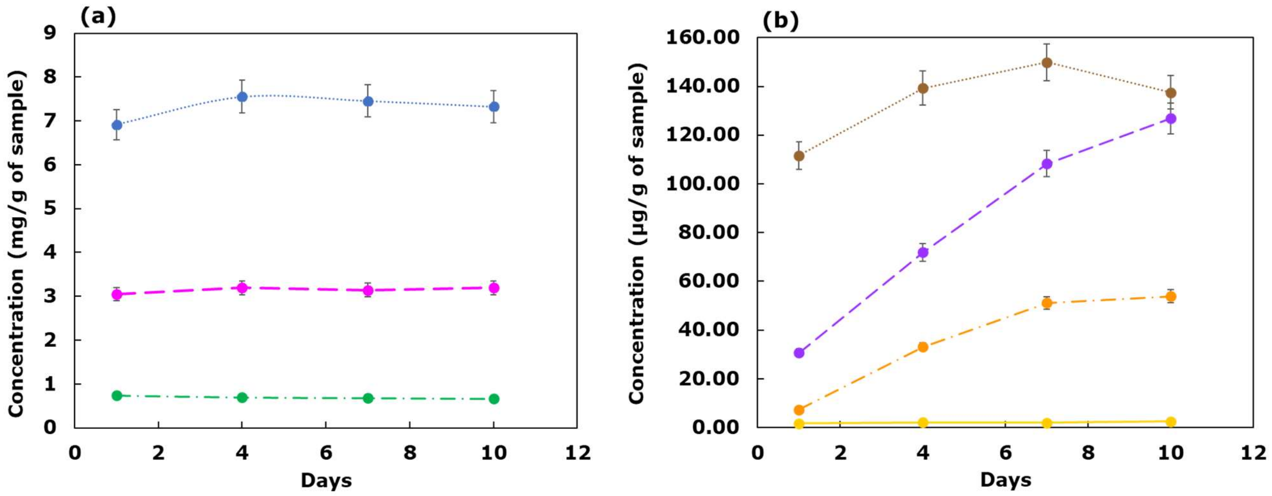

Figure S6. ICP-measured solution concentrations of various elemental species constituting GFC-1 mixture in a 3 m NaOH solution: (a) vanadium (blue dotted line), boron (pink dashed line), zinc (green dash-dotted line), (b) calcium (brown dotted line), silicon (purple dashed line), aluminum (orange dashed-dotted line), and tin (yellow solid line).

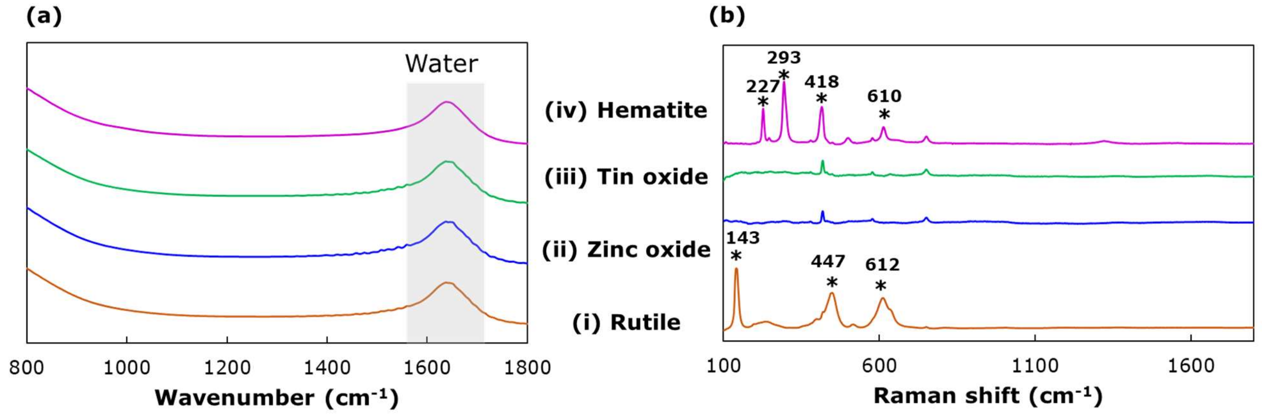

Figure S7. (a) FTIR and baseline corrected (b) Raman spectra of metal oxides GFC components in a basic 3 m NaOH solution. (i) Rutile (TiO_2), (ii) Zinc oxide (ZnO), (iii) Tin oxide (SnO_2), and (iv) Hematite (Fe_2O_3). The Raman peaks corresponding to the solids have been marked with an '*' and their corresponding wavenumbers have been listed.

Section S7. Carbonate Solubility Model

The carbonate anion has two sources in the studied slurries: sodium carbonate (from waste simulants) and lithium carbonate (from solid GFCs) as indicated by Eqn. S7. A single peak, corresponding to CO_3^{2-}, is shared by both components in FTIR. Experiments confirm that CO_3^{2-} anions in solution contributed from either salt are identical. Given only ATR-FTIR spectra as the model input, predictions cannot be made that differentiate the contributions of sodium carbonate and lithium carbonate: only their sum total. However, sodium carbonate and lithium carbonate have different solubilities at the conditions tested. In the studied system, the experimental solubility of lithium carbonate is exceeded, limiting a gravimetric-based calibration procedure.

$$[\mathrm{CO}_3^{2-}]_{(aq)} = [\mathrm{Na}_2\mathrm{CO}_3]_{dissociated} + [\mathrm{Li}_2\mathrm{CO}_3]_{dissociated}$$

$$[\mathrm{Na}_2\mathrm{CO}_3]_{dissociated} = [\mathrm{Na}_2\mathrm{CO}_3]_{added} \qquad \text{(S7)}$$

$$[\mathrm{Li}_2\mathrm{CO}_3]_{dissociated} \neq [\mathrm{Li}_2\mathrm{CO}_3]_{added}$$

Because the PLSR model calibration requires knowledge of the amount of total carbonate dissolved, a solubility model was used to predict the amount of dissolved lithium carbonate (as observed by our ATR-FTIR probe). In lieu of a more complete dissolution model (accounting for all present species), lithium carbonate solubility was estimated by a ternary system comprised of sodium carbonate, lithium carbonate, and 3 m sodium hydroxide solution (NaOH and H_2O).

However, the dissolution of lithium carbonate did not have a significant trend in the process-relevant ranges of sodium carbonate (0.1–1 m). Therefore, a constant lithium carbonate solubility of 0.304 m (the pure component solubility of lithium carbonate in a 3m sodium hydroxide solution at 25 ºC) was applied to training and testing data for the PLSR model where $[\mathrm{Li_2CO_3}]_{added} > [\mathrm{Li_2CO_3}]_{soluble}$. This resulted in the application of the solubility model substitution in 17 of 48 FTIR samples.

The effect of the solubility model was tested in Fig. S8. Use of the solubility model (Fig. S8a) improves the PLSR model performance over quantification lacking a solubility model (Fig. S8b) in terms of both R^2 and visual fit. This indicates that the constant solubility model better matches the data (Fig. S8a) than an assumption of complete dissolution (Fig. S8b).

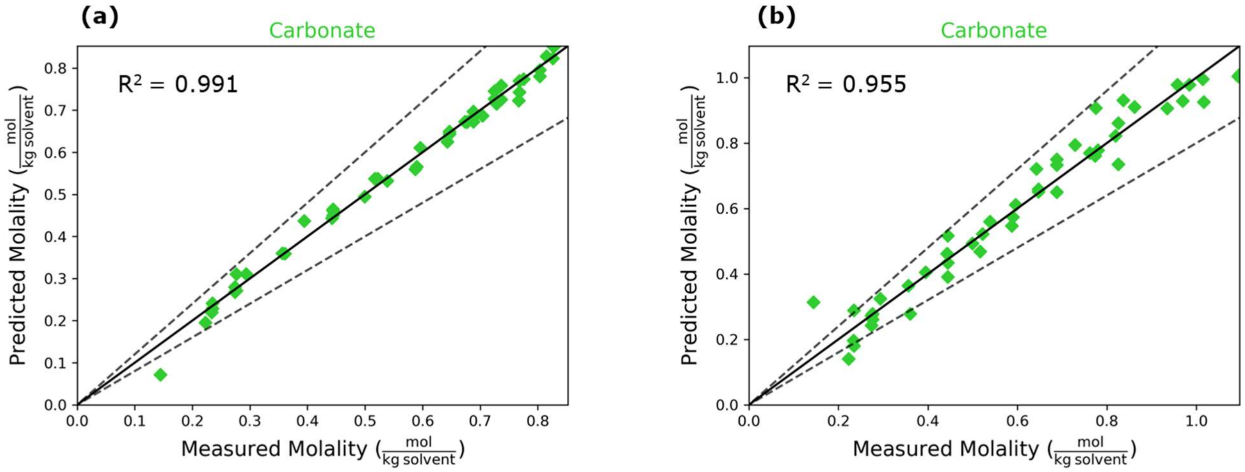

Figure S8. a) Parity plot showing quantification of carbonate with solubility model applied to concentration data, and b) parity plot showing quantification of carbonate with no solubility model applied.

Section S8. Quantification Model Residuals

Distribution of residuals are shown in Fig. S9. Due to the comparatively little sulfate included in these waste simulants, low molality quantification may be hindered by detection limits of sulfate with our ATR-FTIR probe. The calculated LOD is 0.01 mol/kg solvent with a sulfate reference and 12 repeated measurements. Limit of detection was calculated using the method described by Harris.[9] From Fig. S9 we can see that the samples with lower sulfate concentration are close to the limit of detection. In addition, the limit of detection may be further impacted by the overlapping borate peak in all samples, though this is not accounted for in the calculated limit

of detection. For this reason, signal-to-noise ratio may become more important for low-concentration analytes that have overlapping spectral signatures, such as sulfate.

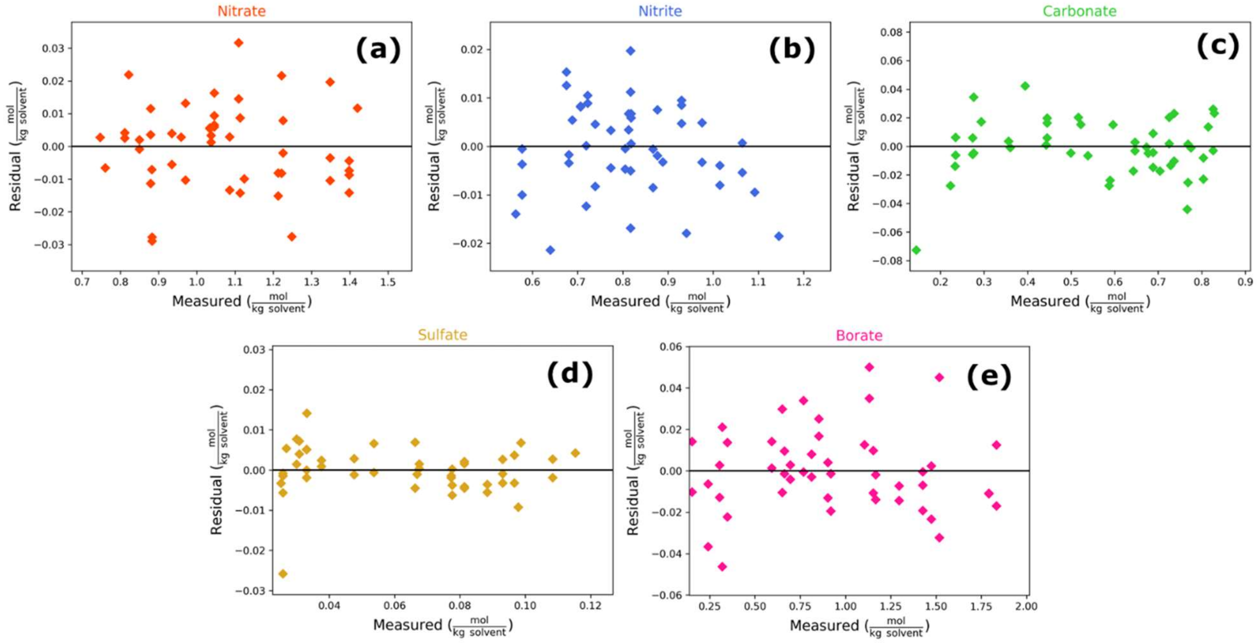

Figure S9. Residual plots of soluble anion quantification using ATR-FTIR spectra input into a PLSR model with 15 latent variables for a) nitrate, b) nitrite, c) carbonate, d) sulfate, and e) borate.

From residual plots for solids (Fig. S10), patterned residuals can be seen at high solids concentrations for all quantified silicates. This might indicate that a PLSR model does not adequately model the relationships between inputs (spectra) and outputs (concentrations) at higher slurry concentrations. Fig. S10 also suggests the nonlinear behavior may not be described by a single function, which would make model corrections component-specific in this regime. For example, the zircon residuals appear to undergo a transition around 25 g solid/kg solvent, where the model begins to underpredict solids concentrations. In contrast, kyanite appears to undergo an analogous transition around 60 g solid/kg solvent. Further analysis is limited by model noise obscuring residual trends.

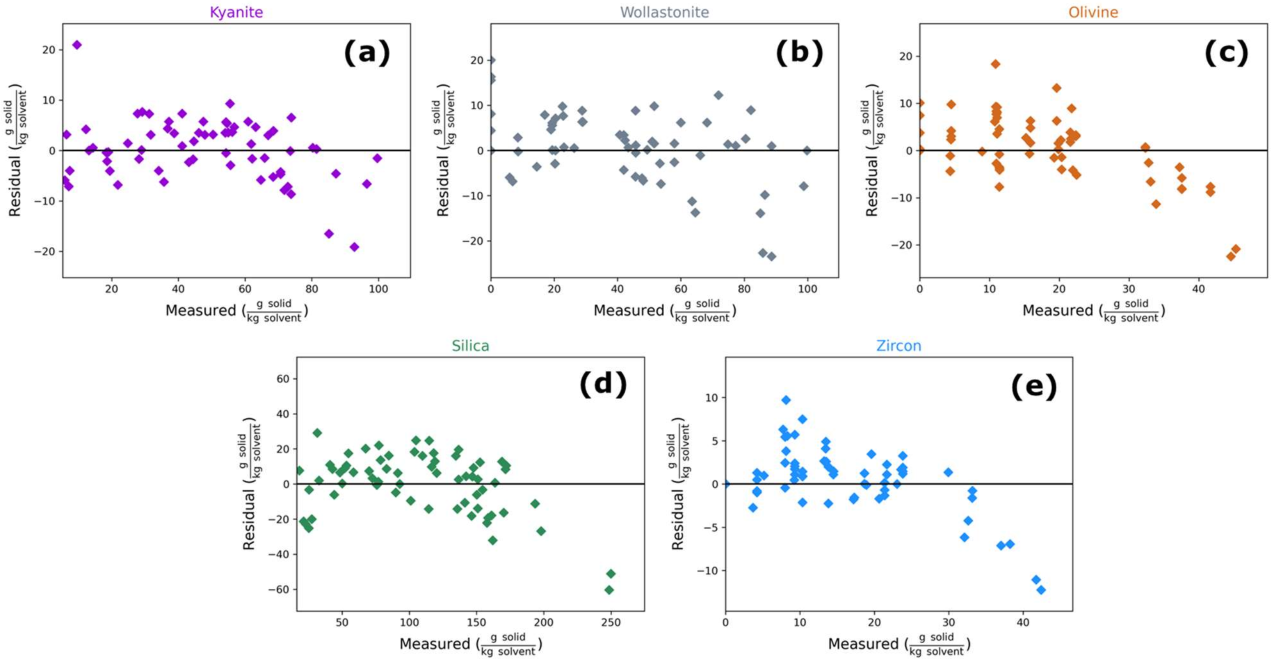

Figure S10. Residual plots of insoluble silicate quantification using Raman spectra input into a PLSR model with 10 latent variables for a) kyanite, b) wollastonite, c) olivine, d) silica, and e) zircon.

Section S9. Independence of Chemical Species in Dataset

Experiments were designed to ensure the components of the dataset was sufficiently independent of all other components. Without sufficient independence between variables, a model may produce unrealistically good quantification. Spurious correlation between variables becomes a greater challenge when working with a high dimensional input space (202 and 3101 features for ATR-FTIR and Raman spectra, respectively), high dimensional output space (5 quantified components for each spectroscopy), and a limited sample space (48 and 66 samples, respectively). An investigation of correlations between outputs (concentration) was performed. The coefficient of determination (the square of correlation, commonly seen as r^2) was used to account for large magnitude correlations. Fig. S9 shows the coefficient of determination of the components. An r^2 value of 1 is expected on the diagonal, because variables are perfectly correlated with themselves.

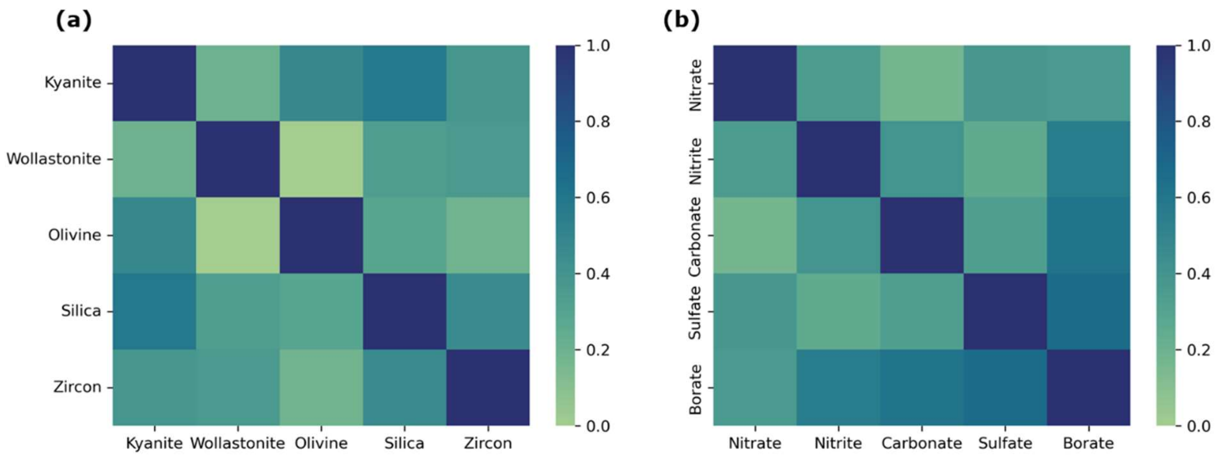

Figure S11. Coefficient of determination plots for five quantified insoluble species (a) and five quantified solution components (b).

The heatmaps in Fig. S11 show the correlation between species for the dataset used in this work. To test the impact of data correlation, two PLSR models were constructed, one corresponding to quantified solids (kyanite, Wollastonite, olivine, silica, and zircon) and the other corresponding to quantified solution species (nitrate, nitrite, carbonate, sulfate, and borate). Each PLSR model was constructed so that four of the respective quantified species were used as model inputs, while the remaining component was quantified. This is structurally similar to the quantification performed in Sections 3.4 and 3.5 except that the input data is the concentrations of other species, rather than spectra. The two PLSR models of this section used four latent variables each, equal to the number maximum allowable with four input variables. This quantification isolates the effects of correlation between species: any prediction capability in Fig. S12 results from correlations in the dataset. If the model is quantifying based on artifacts in the dataset, we expect the quantification accuracy in Fig. S12 to match the quantification accuracy in Fig. 7 and Fig. 9. Fig S12 shows the parity plots for both the soluble species and insoluble species studied.

From the results of the parity plots, it can be seen that a substantial amount of scatter exists for all the species when using other species as model inputs rather than spectra. This indicates that the PLSR models are quantifying the spectra and not making use of spurious correlations in the dataset to produce effective quantification. A weakly positive predictive ability exists for many species. However, this effect appears weak for most species.

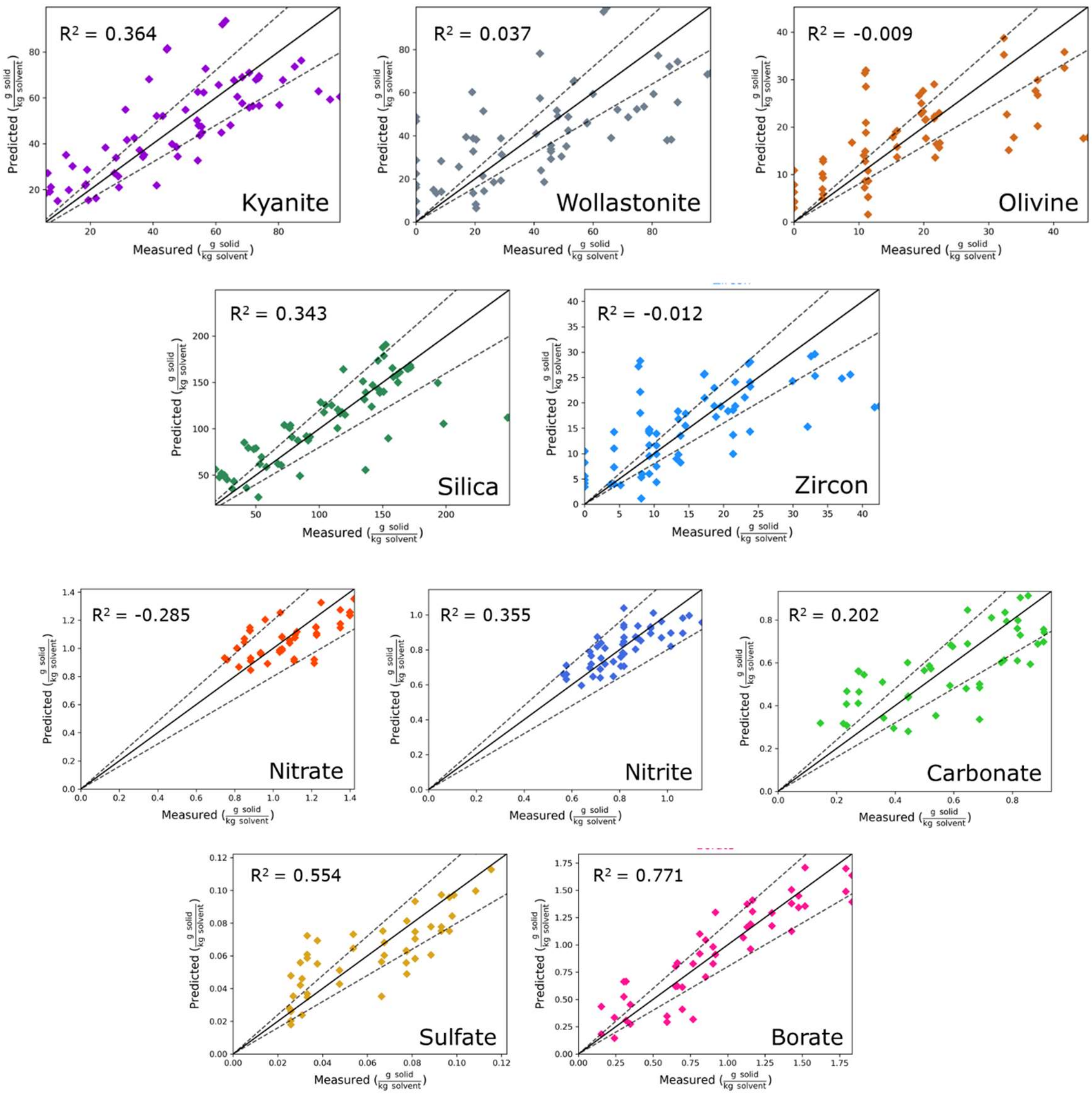

Figure S12. Parity plots for both soluble and insoluble components when using the concentrations of the other species as independent variables.

The high correlation/prediction accuracy of borate in Fig. S12 warrants additional discussion. Borate produces the best quantification of all the species when using concentrations of other species for quantification. This likely results from the manner in which boric acid was added to solutions. Additions were done in two steps: a soluble species addition and a GFC addition. The soluble species addition consisted of nitrate, nitrite, carbonate, and sulfate (in addition to phosphate, acetate, and oxalate as minor species). The GFC addition, however, contained soluble boric acid and lithium carbonate. Because of this, boric acid was added in specific concentrations

dictated by GFC composition rather than designed via pseudorandom uniform distribution like the other species. Despite this, the results in the manuscript with an R^2 value of 0.998 are substantially better than could be achieved through the correlation in Fig. S12.

Supplemental Bibliography:

(1) Russell, R. L.; Schonewill, P. P.; Burns, C. A. Simulant Development for LAWPS Testing; United States. **2017**.

(2) Siegfried, M. J.; Stone, M. E. LAW Simulant Recipes for Evaluation of Real-Time, In-Line Monitoring Instruments. United States. **2020**.

(3) Strachan, C. J.; Rades, T.; Gordon, K. C.; Rantanen, J. Raman Spectroscopy for Quantitative Analysis of Pharmaceutical Solids. *J. Pharm. Pharmacol.* **2007**, *59*, 179–192.

(4) Abdi, H. Partial Least Squares Regression and Projection on Latent Structure Regression (PLS Regression). *Wiley Interdiscip. Rev. Comput. Stat.* **2010**, *2* (1), 97–106.

(5) Wold, S.; Sjöström, M.; Eriksson, L. PLS-Regression: A Basic Tool of Chemometrics. *Chemom. Intell. Lab. Syst.* **2001**, *58* (2), 109–130.

(6) Burnham, K. P.; Anderson, D. R. *Model Selection and Inference: A Practical Information-Theoretic Approach*, 1st ed.; Springer-Verlag, New York, **1998**.

(7) Kocevska, S.; Maggioni, G. M.; Rousseau, R. W.; Grover, M. A. Spectroscopic Quantification of Target Species in a Complex Mixture Using Blind Source Separation and Partial Least-Squares Regression: A Case Study on Hanford Waste. *Ind. Eng. Chem. Res.* **2021**, *60* (27), 9885–9896.

(8) Minnich, C. B.; Helmdach, L.; Ulrich, J.; Feth, M. P. Model-Based Recognition of Mid-Infrared Sensor Fouling in Paracetamol Crystallization. *Chem. Eng. Technol.* **2015**, *38* (8), 1303–1307.

(9) Harris, D. C. *Quantitative Chemical Analysis*, Eighth.; Clancy Marshall, **2010**.
